# Breeding progress and preparedness for mass‐scale deployment of perennial lignocellulosic biomass crops switchgrass, miscanthus, willow and poplar

**DOI:** 10.1111/gcbb.12566

**Published:** 2018-10-23

**Authors:** John Clifton‐Brown, Antoine Harfouche, Michael D. Casler, Huw Dylan Jones, William J. Macalpine, Donal Murphy‐Bokern, Lawrence B. Smart, Anneli Adler, Chris Ashman, Danny Awty‐Carroll, Catherine Bastien, Sebastian Bopper, Vasile Botnari, Maryse Brancourt‐Hulmel, Zhiyong Chen, Lindsay V. Clark, Salvatore Cosentino, Sue Dalton, Chris Davey, Oene Dolstra, Iain Donnison, Richard Flavell, Joerg Greef, Steve Hanley, Astley Hastings, Magnus Hertzberg, Tsai‐Wen Hsu, Lin S. Huang, Antonella Iurato, Elaine Jensen, Xiaoli Jin, Uffe Jørgensen, Andreas Kiesel, Do‐Soon Kim, Jianxiu Liu, Jon P. McCalmont, Bernard G. McMahon, Michal Mos, Paul Robson, Erik J. Sacks, Anatolii Sandu, Giovanni Scalici, Kai Schwarz, Danilo Scordia, Reza Shafiei, Ian Shield, Gancho Slavov, Brian J. Stanton, Kankshita Swaminathan, Gail Taylor, Andres F. Torres, Luisa M. Trindade, Timothy Tschaplinski, Gerald A. Tuskan, Toshihiko Yamada, Chang Yeon Yu, Ronald S. Zalesny, Junqin Zong, Iris Lewandowski

**Affiliations:** ^1^ Institute of Biological, Environmental and Rural Sciences Aberystwyth University Aberystwyth UK; ^2^ Department for Innovation in Biological, Agrofood and Forest systems University of Tuscia Viterbo Italy; ^3^ USDA‐ARS U.S. Dairy Forage Research Center Madison Wisconsin; ^4^ Rothamsted Research Harpenden UK; ^5^ Lohne Germany; ^6^ Horticulture Section, School of Integrative Plant Science Cornell University Geneva New York; ^7^ SweTree Technologies AB Umeå Sweden; ^8^ Institute of Crop Production Ecology Swedish University of Agricultural Sciences Uppsala Sweden; ^9^ INRA‐BIOFORA Orléans France; ^10^ Department of Seed Science and Technology, Institute of Plant Breeding, Seed Science and Population Genetics University of Hohenheim Stuttgart Germany; ^11^ Institute of Genetics, Physiology and Plant Protection (IGFPP) of Academy of Sciences of Moldova Chisinau Moldova; ^12^ INRA‐AgroImpact Péronne cedex France; ^13^ Insitute of Miscanthus Hunan Agricultural University Hunan Changsha China; ^14^ Department of Crop Sciences & Center for Advanced Bioenergy and Bioproducts Innovation, 279 Edward R Madigan Laboratory University of Illinois Urbana Illinois; ^15^ Dipartimento di Agricoltura Alimentazione e Ambiente Università degli Studi di Catania Catania Italy; ^16^ Plant Breeding Wageningen University & Research Wageningen The Netherlands; ^17^ Battersea London UK; ^18^ Julius Kuhn‐Institut (JKI) Bundesforschungsinstitut fur Kulturpflanzen Braunschweig Germany; ^19^ Institute of Biological and Environmental Science University of Aberdeen Aberdeen UK; ^20^ Taiwan Endemic Species Research Institute (TESRI) Nantou County Taiwan; ^21^ Department of Agronomy & The Key Laboratory of Crop Germplasm Resource of Zhejiang Province Zhejiang University Hangzhou China; ^22^ Department of Agroecology Aarhus University Centre for Circular Bioeconomy Tjele Denmark; ^23^ Department of Biobased Products and Energy Crops, Institute of Crop Science University of Hohenheim Stuttgart Germany; ^24^ Department of Plant Sciences, Research Institute of Agriculture & Life Sciences, CALS Seoul National University Seoul Korea; ^25^ Institute of Botany Jiangsu Province and Chinese Academy of Sciences Nanjing China; ^26^ Natural Resources Research Institute University of Minnesota – Duluth Duluth Minnesota; ^27^ Energene sp. z o.o. Wrocław Poland; ^28^ James Hutton Institute University of Dundee Dundee UK; ^29^ GreenWood Resources, Inc. Portland Oregon; ^30^ Hudson‐Alpha Institute for Biotechnology Huntsville Alabama; ^31^ Biological Sciences University of Southampton Southampton UK; ^32^ The Center for Bioenergy Innovation Oak Ridge National Laboratory Oak Ridge Tennessee; ^33^ Field Science Centre for the Northern Biosphere Hokkaido University Sapporo Japan; ^34^ College of Agriculture and Life Sciences 2 Kangwon National University Chuncheon South Korea; ^35^ USDA Forest Service Northern Research Station Rhinelander Wisconsin

**Keywords:** bioenergy, feedstocks, lignocellulose, *M. sacchariflorus*, *M. sinensis*, *Miscanthus*, *Panicum virgatum*, perennial biomass crop, *Populus* spp., *Salix* spp.

## Abstract

Genetic improvement through breeding is one of the key approaches to increasing biomass supply. This paper documents the breeding progress to date for four perennial biomass crops (PBCs) that have high output–input energy ratios: namely *Panicum virgatum* (switchgrass), species of the genera *Miscanthus* (miscanthus), *Salix* (willow) and *Populus* (poplar). For each crop, we report on the size of germplasm collections, the efforts to date to phenotype and genotype, the diversity available for breeding and on the scale of breeding work as indicated by number of attempted crosses. We also report on the development of faster and more precise breeding using molecular breeding techniques. Poplar is the model tree for genetic studies and is furthest ahead in terms of biological knowledge and genetic resources. Linkage maps, transgenesis and genome editing methods are now being used in commercially focused poplar breeding. These are in development in switchgrass, miscanthus and willow generating large genetic and phenotypic data sets requiring concomitant efforts in informatics to create summaries that can be accessed and used by practical breeders. Cultivars of switchgrass and miscanthus can be seed‐based synthetic populations, semihybrids or clones. Willow and poplar cultivars are commercially deployed as clones. At local and regional level, the most advanced cultivars in each crop are at technology readiness levels which could be scaled to planting rates of thousands of hectares per year in about 5 years with existing commercial developers. Investment in further development of better cultivars is subject to current market failure and the long breeding cycles. We conclude that sustained public investment in breeding plays a key role in delivering future mass‐scale deployment of PBCs.

## 1 INTRODUCTION

Increasing sustainable biomass production is an important component of the transition from a fossil fuel‐based economy to renewables. Taking the United Kingdom as an example, Lovett, Sünnenberg, and Dockerty (2014) suggested that 1.4 million ha of marginal agricultural land could be used for biomass production without compromising food production. Assuming a biomass dry matter (DM) yield of 10 Mg/ha and a calorific value of 18 GJ/Mg DM, 1.4 million ha would deliver around 28 TWh of electricity (with 40% biomass conversion efficiency) which would be ~8% of primary UK electricity generation (336 TWh in 2017 (DUKES, 2017)). To achieve this by 2050, planting rates of ~35,000 ha/year would be needed from 2022, in line with calculations by Evans (2017). The current annual planting rates in the United Kingdom are orders of magnitude short of these levels at only several hundred hectares per year. Similar scenarios have been generated for other countries (BMU, 2009; Scarlat, Dallemand, Monforti‐Ferrario, & Nita, 2015).

If perennial biomass crops (PBCs) are to make a real contribution to sustainable development, they should be grown on agricultural land which is less suitable for food crops (Lewandowski, 2015). This economically “marginal” land is typically characterized by abiotic stresses (drought, flooding, stoniness, steep slope, exposure to wind and sub‐optimal aspect), low nutrients and/or contaminated soils (Tóth et al., 2016). In these challenging environments, PBCs need resilience traits. They also need high output:input ratios for energy (typically 20–50) to deliver large carbon savings. Land may also be marginal due to environmental vulnerability. Much of the value for society from the genetic improvement of these crops depends on positive effects arising from highly productive perennial systems. In addition to producing biomass as a carbon source to replace fossil carbon, these crops reduce nitrate leaching (Pugesgaard, Schelde, Larsen, Lærke, & Jørgensen, 2015), making them good candidates to help fulfil Water Framework Directive (2000/60/EC) and can increase soil carbon storage during their production (McCalmont et al., 2017).

The objective of this paper was to report on the preparedness for wide deployment by summarizing the technical state of the art in breeding of four important PBCs: namely switchgrass, miscanthus, willow and poplar. These four crops are the most promising and advanced PBCs for temperate regions and have therefore the focus here. Switchgrass and miscanthus are both rhizomatous grasses with C_4_ photosynthesis, while willow and poplar are trees with C_3_ photosynthesis. Specifically, this paper (a) reviews available crop trait genetic diversity information; (b) assesses the progress of conventional breeding technologies for yield resilience and biomass quality; (c) reports on progress with new molecular‐based breeding technologies to increase speed and precision of selection; and (d) discusses the requirements and next steps for breeding of PBCs, including commercial considerations in order to sustainably meet the biomass requirements of a growing worldwide bioeconomy.

**Figure 1:**
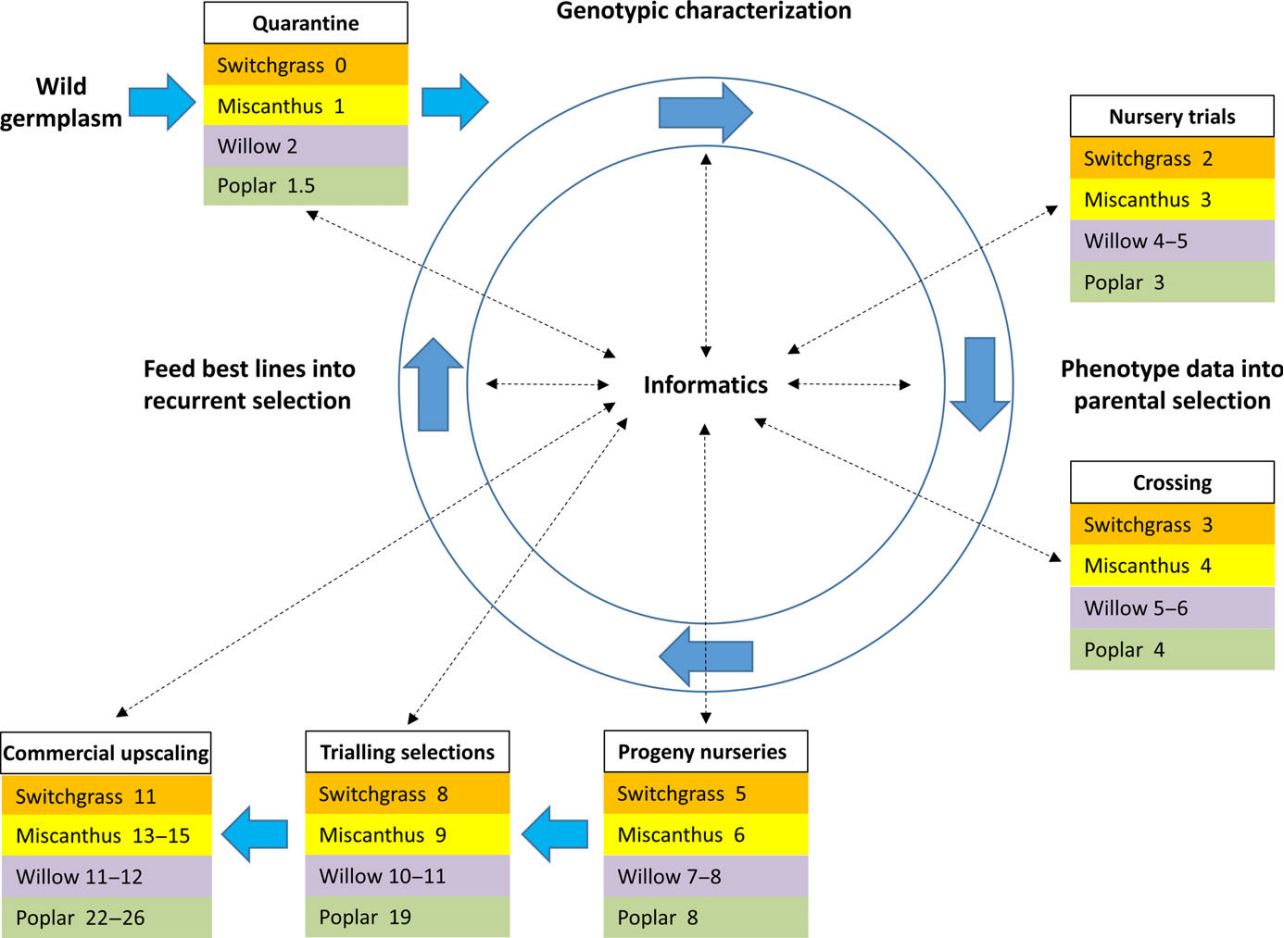
Cumulative minimum years needed for the conventional breeding cycle through the steps from wild germplasm to the commercial hybrids in switchgrass, miscanthus, willow and poplar. Information links between the steps are indicated by dotted arrows and highlight the importance of long‐term informatics to maximize breeding gain

We summarize the crop‐specific attributes, the location of breeding programmes, the current availability of commercial cultivars and yield expectations in selected environments (Table 1), and the generalized breeding targets for all PBCs (Table 2). Economic information relating to the current market value of the biomass and the investment in breeding are presented for different countries/regions in Table 3. We also present a comparison of the prebreeding and conventional breeding efforts step‐by‐step, starting with wild germplasm collection and evaluation before wide crossing of wild relatives (Table 4). Hybridization is followed by at least 6 years of selection and evaluation before commercial upscaling can begin (Figure 1). Recurrent selection, often over decades, is used within parent populations as part of an ongoing long‐term process to produce hybrid vigour (Brummer, 1999). In the following sections, the state of the art and new opportunities of breeding switchgrass, miscanthus, willow and poplar are described. The application of modern breeding technologies is compared for the four crops in Table 5. It is most advanced in poplar and is therefore described in most detail.

**Table 1:** Breeding‐related attributes for four leading perennial biomass crops (PBCs)

Species	Switchgrass	Miscanthus	Willow	Poplar
Type	C4—Grass	C4—Grass	C3—SRC	C3—SRC/SRF
Sources of indigenous germplasm	CA[Link] to MX, east of Rocky Mountains.	Eastern Asia and Oceania	Predominantly Northern hemisphere	Northern hemisphere
Breeding system	Monoecious, outcrossing	Monoecious, outcrossing	Dioecious, outcrossing	Dioecious, outcrossing
Ploidy	4×, 8×	2×, 3×, 4×	2×−12×	2×
Species within *genus*	~450	~14	~400	~30–32
Types used mainly for breeding	US: Lowland ecotype (subtropical climates) and upland ecotype (temperate climates)	EU[Link], JP, SK and US: *M.sin.* and *M.sac*.	EU: *S. viminalis *× *schwerinii*,* S. dasyclados *× *rehderiana, S. dasyclados, S. viminalis* US and UK: *S. viminalis *× *miyabeana, S. miyabeana* US: *S. purpurea *× *miyabeana, S. purpurea*	*Populus trichocarpa, P. deltoides, P. nigra, P. suaveolens* subsp.* maximowiczii, P. balsamifera P. alba* and hybrids
Typical haploid genome size (Mbp)	~1,500	*M.sin. ~5,400 and M.sac. ~4,400* (Rayburn, Crawford, Rayburn, & Juvik, 2009)	~450	~485 ± 10
Breeding programmes	CA: 2000 (REAP, Quebec) US: 1992 (Nebraska 1992 (Oklahoma) 1992 (Georgia) 1996 (Wisconsin) 1996 (South Dakota) 2000 (Tennessee) 2002 (Mississippi) 2007 (Oklahoma) 2008 (Rutgers, New Jersey) 2012 (Cornell, New York) 2012 (Urbana‐Champaign, Illinois)	DE: 1990s (Klein‐Wanzleben) NL: 2000s (Wageningen) UK: 2004 (Aberystwyth) CN: 2006 (Changsha) JP: 2006 (Hokkaido) US: 2006 (California—CERES Inc.) 2006 (California and Indiana—MBI) 2008 (Urbana‐Champaign) SK: 2009 (Suwon‐SNU) and (Muan, NICS of RDA) FR: 2011 (Estrées‐Mons)	UK: 1980s (Long Ashton, relocated to Rothamsted Research in 2002) SE: 1980s (Svalöf Weibull/Salixenergi Europa AB) US: 1990s (Cornell, New York) PL: 2000s (Olsztyn, University of Warmia and Mazury)	SE: 1939 (Mykinge, Ekebo Research Institute) 1990s (Uppsala, SLU) 2010 (Uppsala, STT) US: 1927 (New York, Oxford Paper Company and New York Botanical Garden, Wheeler et al., 2015) 1979 (Washington, UW and Oregon, GWR) 1980–1995 (Mississippi, MSState and GWR) 1996 (Minnesota, UMD NRRI and GWR) IT: 1983 (Piedmont, AFV) FR: 2001 (Orléans & Nancy, INRA; Charrey‐sur‐Saône & Pierroton, FCBA; Nogent‐sur‐Vernisson, IRSTEA) DE: 2008 (Göttingen, NW‐FVA)
Current commercial varieties on the market	US: No commercial hybrids	CA: 2 EU: 1 (*M *× *g* from different origins) + selected *M.sin.* for thatching in DK US: 3 (but 2 are genetically identical)	UK: 25 US: 8	EU: DE: <10, FR: 44, IT: 10–15, SE: ~14 US: 8–12 Southeast, 8–14 Upper Midwest, 10 Pacific Northwest
Precommercial cultivars expected to be on the market in 3 years	US: 36 registered cultivars (half are random seed increases from natural prairies, and half are bred varieties); Most are public releases; few are protected, patented or licensed	NL: 8, seeded hybrids, van Dinter Semo, MTA UK: 4, seeded hybrids, CERES (Land O'Lakes) and Terravesta Ltd., MTA US: None FR: None	EU: 53 registered with CPVO for PBR UK: 20 registered with CPVO for PBR US: 18 clones from Cornell in multisite trials	CA: unquantified, UAlberta and Quebec FR: 8–12 SRF clones, licence‐based IT: 5 SRC and 6 SRF, AFV, MTA or licence SE: 14, STT, licence‐based and MTA US: ~19 Southeast; 5–7 Upper Midwest from UMD NRRI, MTA; 6–12 North Central from GWR, MTA; Pacific Northwest: 8 clones, GWR, MTA; 24 in multilocation yield trials, GWR
Commercial yield (t DM ha^−1^ year^−^^1^)	US: 3–18 EU: 8–12	CN: 20 – 30 US: 10– 25 EU: 7 – 20	UK: 8–14 US: 8–14	EU: 5–20 (SE and Baltic Countries: 8–12) US: 10–22 (10–12 North Central, 12–16 Southeast, 15–22 Pacific Northeast)
Harvest rotation and commercial stand lifespan	Annual for 10–12 years	Annual for 10–25 years (*M.sac* has been used for ~30 years in China)	2‐ to 4‐year cycle for 22–30 years	SRC: 3‐ to 7‐year cycle for 20 years SRF: 10‐ to 12‐year cycle for >50 years
Adaptive range	Open‐pollinated and synthetic cultivars are limited in adaptation by temperature and precipitation (~8 breeding zones in US and CA)	Standard *M *× *g* is widely adapted in EU, but is limited in the United States by insufficient winter hardiness for Northern Midwest and heat intolerance in the south. Novel *M.sac*. × *M.sin.* hybrids and *M.sin*. × *M.sin.* hybrids selected in continental DE have shown a wide adaptive range in EU (Kalinina et al., 2017). Ongoing trials on heavy metal contaminated soils indicate tolerance by exclusion (Krzyżak et al., 2017)	Different hybrids are needed for different zones Best hybrids show some G × E (US), and some hybrids low G × E (Fabio et al., 2017)	Different hybrids are needed for different climatic zones. Hybrids that are adapted for growing seasons of ~6 months and relatively short days in Southern Europe are maladapted to short growing seasons of ~4 months and relatively long days in Northern EU The most broadly adapted varieties come from the *P. canadensis* taxon

Note

AFV: Alasia Franco Vivai; Cornell: Cornell University; CPVO: Community Plant Variety Office; FCBA: Forest, Cellulose, Wood, Construction and Furniture Technology Institute; G × E: genotype‐by‐environment interaction; GWR: GreenWood Resources; INRA: French National Institute for Agricultural Research; IRSTEA: National Research Unit of Science and Technology for Environment and Agriculture; *M. sac*:* M. sacchariflorus and M × g (M. × giganteus*); *M. sin*:* Miscanthus sinensis*; MBI: Mendel Biotechnology Inc.; Mbp: mega base pair; MSState: Mississippi State University; MTA: material transfer agreement; NICS: National Institute of Crop Science; NW‐FVA: Northwest German Forest Research Institute; PBR: plant breeder's right; RDA: Rural Development Administration; REAP: Resource Efficient Agriculture Production; SLU: Swedish University of Agricultural Sciences; SNU: Seoul National Uni.; SRC: short‐rotation coppice; SRF: short‐rotation forestry; STT: SweTree Tech; t DM ha^−1^ year^−1^: tons of dry matter per hectare per year; UAlberta: University of Alberta; UMD NRRI: University of Minnesota Duluth's Natural Resources Research Institute; UMN: University of Minnesota; UW: University of Washington; UWM: University of Warmia and Mazury.

ISO Alpha‐2 letter country codes.

EU is used for Europe.

**Table 2:** Generalized improvement targets for perennial biomass crops (PBCs)

Net energy yield per hectare
Increased yield
Reduced moisture content at harvest
Physical and chemical composition for different end‐use applications
Increased lignin content and decreased corrosive elements for thermal conversion
Reduced recalcitrance through decreased lignin content and/or modified lignin monomer composition to reduce pretreatment requirements for next‐generation biofuels by saccharification and fermentation
Plant morphological differences which influence biomass harvest, transport and storage (e.g., stem thickness)
Propagation costs
Improved cloning systems (trees and grasses)
Seed systems (grasses)
Optimizing agronomy for each new cultivar
Resilience through enhanced
Abiotic stress tolerance/resistance (e.g., drought, salinity, and high and low temperature )
Biotic stress resistance (e.g., insects, fungal, bacterial and viral diseases)
Site adaptability especially to those of marginal/contaminated agricultural land

**Table 3:** Preparedness for mass upscaling: current market value and research investment in four perennial biomass crops (PBCs)

Species	Switchgrass	Miscanthus	Willow	Poplar
Current commercial planting costs per ha	US[Link]: 200 USD[Link] in the establishment year	DE: 3,375 Euro[Link] (Xue, Kalinina, & Lewandowski, 2015) UK: 2,153 GBP[Link] (Evans, 2016) reduced to 1,169 GBP with *M*x*g* rhizome where the farmer does the land preparation ( www.terravesta.com) US: 1,730–2,225 USD	UK: 1,500–1,739 GBP plus land preparation (Evans, 2016) US: 1,976 USD (= 800 USD/acre)	IT: 1,100 Euro FR/SE: 1,000–2,000 Euro US: 863 USD/ha (Lazarus, Headlee, & Zalesny, 2015)
Current market value of the biomass per t DM	US: 80–100 USD	UK: ~80 GBP (bales) (Terravesta, personal communication) US: 94 USD (chipped)	UK: 49.41 GBP (chipped) (Evans, 2016) US: 55–70 USD	IT: 100 Euro US: 80–90 USD delivered based on 40 miles of haulage
Science for genetic improvement: projects in the last 10 years	US:>50 projects funded by US DOE and USDA NIFA	CN: >30 projects funded by CN‐NSFC and MBI EU[Link]: 6 projects (OPTIMISC, OPTIMA, WATBIO, SUNLIBB, FIBRA and GRACE) FR: BFF SK: 2 projects funded by IPET and PMBC US: EBI, CABBI, multiple DOE Feedstock Genomics and USDA AFRI projects	UK: BSBEC, BEGIN (2003–2010) US: US DOE JGI Genome Sequencing (2009–2012; 2015–2018); USDA Northeast Sun Grant (2009–2012); USDA NIFA NEWBio Consortium (2012–2017); USDA NIFA Willow SkyCAP (2018–2021)	EU: FP7 (Energy Poplar, NovelTree, WATBIO, Tree4Future) SE: Climate‐adapted poplars and Nanowood, SLU and STT US: Bioenergy Science (ORNL), USDA feedstocks genomics programme, BRDI and BRC‐CBI
Major projects supporting crossing and selection cycles in the last 10 years	US and CA: 12 projects	NL: RUE miscanthus, PPP, 2015–2019, 50–100 K Euro/year UK: GIANT‐LINK, PPI, 2011–2016, 1.3 M GBP/year US: EBI/CABBI, ~0.5 M USD/year	UK: BEGIN 2000–2010 US: USDA Northeast Sun Grant (2009–2012); USDA NIFA NEWBio Consortium (2012–2017); USDA NIFA Willow SkyCAP (2018–2021)	FR: 14 regional and national projects, 100–150 k Euro/year SE: STT: one breeding effort as an internal project in 2010 with a resulting progeny trial US: DOE Sun Grant feedstock development partnership programme (including Willow)
Current annual investment in projects for translation into commercial hybrids	US:>20 M USD/year	UK: MUST, 2016–2019, 0.5 M GBP/year US: CABBI/USDA AFRI, 2017–2022 0.5 M USD/year	US: USDA NIFA NEWBio ~0.4 M USD/year	FR: Science for improvement: ~20 k Euro/year US: USDA under AFRI Co‐ordinated Ag. Producer projects. And several translational genomics projects without public funding.
Upscaling time (years) to produce sufficient propagules to plant>100 ha	US: Using seed 2–3 years	UK: Rhizome for 1–2,000 hectares can be ready in 6 months UK/NL: 20 ha of seed hybrids planted in 2018. In 2019, sufficient seed for 50–100 ha is expected	UK: 3 years using conventional cuttings, faster using micropropagation	FR, IT: 3 years by vegetative propagation SE: >3 years by vegetative propagation (cuttings) and 3 years by micropropagation

Note

AFRI: Agriculture and Food Research Initiative in the United States; BEGIN: Biomass for Energy Genetic Improvement Network; BFF: Biomass For the Future; BRC‐CBI: Bioenergy Research Centre‐Centre for Bioenergy Innovation; BRDI: Biomass research development initiative; BSBEC: BBSRC Sustainable Bioenergy Centre; CABBI: Center for advanced bioenergy and bioproducts innovation in the United States; CN‐NSFC: Natural Science Foundation of China; DOE: Department of Energy in the United States; EBI: Energy Biosciences Institute; FIBRA: Fibre crops as sustainable source of biobased material for industrial products in Europe and China; FP7: Seventh Framework Programme in the EU; GIANT‐LINK: Genetic improvement of miscanthus as a sustainable feedstock for bioenergy in the United Kingdom; GRACE: GRowing Advanced industrial Crops on marginal lands for biorEfineries; IPET: Korea Institute of Planning and Evaluation for Technology in Food, Agriculture, Forestry and Fisheries; JGI: Joint Genome Institute; MBI: Mendel Biotechnology Inc.; MUST: Miscanthus UpScaling Technology; NEWBio: Northeast Woody/Warm‐season Biomass Consortium; NIFA: National Institute of Food and Agriculture in the United States; NovelTree: Novel tree breeding strategies; OPTIMA: Optimization of perennial grasses for biomass production in the Mediterranean area; OPTIMISC: Optimizing bioenergy production from Miscanthus; ORNL: Oak Ridge National Lab; PMBC: Plant Molecular Breeding Center of the Next Generation Biogreen Research Centers of the Republic of Korea; PPI: public–private investment; PPP: public–private partnership; RUE: radiation use efficiency; SkyCAP: Coordinated Agricultural Project; SLU: Swedish University of Agricultural Sciences; STT: SweTree Tech; SUNLIBB: Sustainable Liquid Biofuels from Biomass Biorefining; Tree4Future: Designing Trees for the future; USDA: US Department of Agriculture; WATBIO: Development of improved perennial nonfood biomass and bioproduct crops for water stressed environments.

ISO Alpha‐2 letter country codes.

Local currencies are used as at 2018: Euro, GBP (Great Britain Pound); USD (US Dollar).

EU is used for Europe.

**Table 4:** Prebreeding research and the status of conventional breeding in four leading perennial biomass crops (PBCs)

Breeding technology step	Use	Prerequisite steps	Limitations	Switchgrass	Miscanthus	Willow	Poplar
1. Collected wild accessions or secondary sources available for breeding	Provide a broad base of useful traits	Respect for CBD and Nagoya protocol on collections after 2014	Not all indigenous genetic resources are accessible under CBD for political reasons	US[Link]: ~2,000 (181 in official GRIN gene bank of which 96 were available; others in various unofficial collections)	CN: Changsha, ~1,000 and Nanjing, 2,000 from China DK: ~120 from JP FR: working collection of ~100 (mainly *M sin*) JP: ~1,500 from JP, SK and CN NL: working collection of ~300 *M.sin*. SK: ~700 from SK, CN, JP and RU UK: ~1,500 accessions (from ~500 sites) of which 1,000 are in field nurseries in 2018 US: 14 in GRIN global US: Illinois, ~1,500 collections made in Asia with 25% currently available in United States for breeding	UK: 1,500 with about 20 in common with the United States US: 350 largely unique	FR: 3,370 *Populus deltoides* (650), *P. nigra* (2,000), *P. trichocarpa* (600) and *P. maximowiczii* (120) IT: 30,000, *P. deltoids, P. nigra, P*.* maximowiczii and P. trichocarpa* SE: 13,000 accessions, mainly *P. trichocarpa,* by STT and SLU US: GWR: 150 *P. trichocarpa* and 300 *P*.* maximowiczii* accessions for Pacific Northwest program; 535 *P. deltoides* and ~200 *P. nigra* accessions for Southeast programme and 77 *P. simonii* from Mongolia UMD NRRI: 550+ clonal accessions (primarily *P. *× *canadnesis,* along with *P. deltoides* and *P. nigra* parents) for Upper Midwest program
2. Wild accessions which have undergone phenotypic screening in field trials	Selection of parental lines with useful traits	Strong partnerships to run multilocation field trials to phenotype consistently the accessions/genotypes in different environments and databases	Cost of running multilocation trials	US: >500,000 genotypes	CN: ~1,250 in Changsha, ~1,700 in Hunan, Jiangsu, Shandong, Hainan NL: ~ 250 genotypes JP: ~1,200 SK: ~400 UK‐led: ~1,000 genotypes in a replicated trial US‐led: ~1,200 genotypes in multi‐location replicated trials in Asia and North America: *M.sin*.: SK, CN, JP, CA (Ontario), US (Illinois and Colorado); *M.sac*.: SK (Kangwon), CN (Zhejiang), JP (Sapporo), CA (Onatario), US (Illinois), DK (Aarhus)	UK: >400 US: 180	FR: 2,720 IT: 10,000 SE: ~150 US: GWR: ~1,500 wild *P. trichocarpa* phenotypes and OP seedlots (total of 70) from the *P. maximowiczii* species complex (*suaveolens*,* cathayana*,* koreana*,* ussuriensis*,* maximowiczii*) and 2,000–3,000 *P. deltoides* collections (based on 104s‐generation families) UMD NRRI: screened seedlings from a *P. deltoides* (OP or CP) breeding programme (1996–2016) >7,200 OP seedlings of *P. nigra* under Minnesota climatic conditions (improve parental population)
3. Wild germplasm genotyping	Construct phylogenetic trees and dissimilarity indices	A managed living collection of clonal types	The type of molecular analysis—AFLP, cpDNA, RADseq, whole genome sequencing	US: ~20,000 genotypes	CN: Changsha, ~1,000; Nanjing, 37 FR: 44 by cpDNA (Feng et al., 2014) JP: ~1,200 NL: 250 genotypes by RADseq UK: ~1,000 by RADseq SK: ~300 US: Illinois: RADseq on all wild accessions.	UK: ~400 US: 225	FR: 2,310 SE: 150 US: 1,500
4. Exploratory crossing and progeny tests	Discover good parental combinations—general combining ability	Geographic separation, species or phylogenetic trees	Costly long‐term multi‐location trials for progeny	US: >10,000	CN: ~10 FR: ~200 JP: ~30 NL: 3,600 SK: ~20 UK: ~4,000 (~1,500 *M.sin*.) US: 500–1,000	UK: >700 since 2003 >800 EWBP (1996–2002) US: 800	FR: Cloned 13 × 13 factorial mating design with 3 species [10 *P. deltoides*, 8 *P. trichocarpa*, 8 *P. nigra*) US: GWR: 1,000 exploratory crossings of *P. fremontii*,* P. simonii* for improved drought tolerance UMD NRRI: crossed native *P. deltoides* with non‐native *P. trichocarpa* and *P. maximowiczii*. Majority of progeny populations suffered from climatic and/or pathogenic susceptibility issues
5. Wide intraspecies hybridization	Discover good parental combinations—general combining ability	1 to 4 above, informatics	Flowering synchronization and low seed set	US: ~200	CN: ~10 NL: 1,800 SK: ~10 UK: ~500	UK: 276 US: 400	IT: 500 SE: 120 US: 50–100
6. Within species recurrent selection	Concentration of positive traits	Identification of the right heterotic groups in steps 1–5	Difficult to introduce new germplasm without dilution of best traits	US: >40 populations undergoing recurrent selection	NL: 2 populations UK: 6 populations US: ~12 populations to be established by 2019	UK: 4 US: 150	FR: *P. deltoides* (>30 FS families – 900 clones), *P. nigra* (>40 FS families – 1,600 clones) and *P. trichocarpa* (15 FS Families −350 clones) IT: 80 SE: ca. 50 parent breeding populations within *P. trichocarpa* US: Goals is ~100 parent breeding populations within *P. trichocarpa*,* P*.* deltoides*,* P. nigra* and *P. maximowiczii*
7. Interspecies hybrid breeding	Combining complementary traits to produce good morphotypes and heterosis effects	All the steps 1–6	In early stage, improvements are unpredictable. A wide base is costly to manage	None	CN: Changsha, 3; Nanjing, 120 *M.sin. *× *M. flor*. JP: ~5 with *Saccharum spontaneum* SK: 5	UK: 420 US: 250	FR:*P. canadensis* (1800), *P. deltoides *× *P. trichocarpa* (800) and *P. trichocarpa* × *P. maximowiczii* (1,200) IT: ~120 US: 525 genotypes in eastside hybrid programme, 205 westside hybrid programme and ~1,200 in Southeast *P. deltoides* program
8. Chromosome doubling	A route to triploid seeded types, doubling a diploid parent of known breeding value from recurrent selection	Needs 1–7 to help identify the right parental lines	Doubled plants are notorious for reverting to diploid	US: ~50 plants taken from tetraploid to octaploid, which are now in field trials	CN: ~10 *M.sac*. triploids DK: Several attempts in mid 90s FR: 5 genotypes of M *sin* UK: 2 *M.sin., 1 M.flor.* and 1 *M.sac*. US: ~30	UK: Attempted but not routinely used US: Not part of the regular breeding because there exist natural polyploids	*N*/A
9. Double Haploids	A route to producing homogeneous progeny and also for the introduction of transgenes or for genome editing	Needs 1–7 to help identify the right parental lines	Fully homozygous plants are weak and easily die, and may not flower synchronously	US: None yet attempted, due to poor vigour and viability of haploids	CN: 2 genotypes of *M.sin and M.flor* UK: Dihaploids of *M.sin, M.flor* and *M.sac.* and *2 transgenic M.sin.* via anther culture US: 6 created and used to identify miss‐identifying paralogue loci (caused by recent genome duplication in miscanthus). Used in creating the reference genome for *M.sin*. Very weak plants and difficult to retain the lines	*N*/A	*N*/A
10. Embryo rescue	An attractive technique for recovering plants from sexual crosses		The majority of embryos cannot survive in vivo or become long‐time dormant	None	UK: one 3× hybrid	UK: Embryos rescue protocol proved robust at 8 days post‐pollination	FR: An embryo rescue protocol proved robust and improved hybridization success for *P. deltoides* × *P. trichocarpa* crosses
11. Pollen storage	Flexibility to cross interesting parents without need of flowering synchronization			None	No success to date, notoriously difficult with grasses including sugarcane	Fresh pollen commonly used in crossing Pollen storage in some interspecific crosses	FR: Both stored (cryobank) and fresh individual pollen

Note

AFLP: amplified fragment length polymorphism; CBD: convention on biological diversity; CP: controlled pollination; CpDNA: chloroplast DNA; EWBP: European willow breeding programme; FS: full‐sib; G × E: genotype‐by‐environment; GRIN: germplasm resources information network; GWR: GreenWood Resources; *M.flor*.: *Miscanthus floridulus*;* M.sac*.: *M. sacchariflorus*;* M.sin.: M. sinensis*;* N*/A: not applicable; NRRI: Natural Resources Research Institute; OP: open pollination; RADseq: restriction site‐associated DNA sequencing; SLU: Swedish University of Agricultural Sciences; STT: SweTree Tech; UMD: University of Minnesota Duluth.

ISO Alpha‐2 letter country codes.

**Table 5:** Status of modern plant breeding techniques in four leading perennial biomass crops (PBCs)

Breeding technology	Use	Prerequisite steps	Limitations	Switchgrass	Miscanthus	Willow	Poplar
MAS	Use of marker sequences that correlate with a trait allowing early progeny selection or rejection/locating known genes for useful traits (such as height) from other species in your crop	Breeding programme relevant biparental crosses (Table 4) to create “mapping populations” and QTL identification and estimation to identify robust markers; alternatively, a comprehensive panel of unrelated genotypes for GWAS	Ineffective where traits are affected by many genes with small effects	US: Literally dozens of studies to identify SNPs and markers of interest. No attempts at MAS as yet, largely due to population specificity	CN: SSR, ISSR markers for *M.sin*., *M.sac*. *and M.flor*. FR: 2 *M.sin* mapping populations (BFF project) NL: 2 *M.sin* mapping populations (Atienza, Ramirez, et al., 2003; Atienza, Satovic, Petersen, Dolstra, & Martin, 2003; Van Der Weijde et al., 2017a; Van der Weijde, Huxley, et al., 2017; Van der Weijde, Kamei, et al., 2017; Van der Weijde, Kiesel, et al., 2017) SK: 2 GWAS panels (1 collection of *M. sin*, 1 diploid biparental F_1_ population) in the pipeline UK: 12 families to find QTL in common in different families of the same and different species and hybrids US: 2 large GWAS panels, 4 diploid bi‐parental F_1_ populations, 1 F_2_ population (additional in pipeline)	UK: 16 families for QTL discovery, 2 crosses MAS screened, 1 potential variety selected US: 9 families, genotyped by GBS (8 are F_1_, one is F_2_), a number of promising QTL, development of markers in progress.	FR (INRA): tested in large FS families and in factorial mating design, low efficiency due to family specificity US: 49 families
GS	Method to accelerate breeding through reducing the resources for cross attempts by predicting the performance of progeny of crosses (and in research to predict best parents to use in biparental crosses)	A “training population” of individuals from stages above that have been both genotyped and phenotyped to develop a model that takes genotypic data from a “candidate population” of untested individuals and produces GEBVs (Jannink, Lorenz, & Iwata, 2010)	Risks of poor prediction. Progeny testing needs to be continued during the time the training set is being phenotyped and genotyped. In this time, next‐generation germplasm	US: One programme so far—USDA in Wisconsin. Three cycles of genomic selection completed and ready to begin the second round of training and recalibration of genomic prediction models	CN: ~1,000 genotypes NL: preliminary models for *M.sin* developed UK: ~1,000 genotypes US: Preliminary models for *M.sac* and *M.sin* developed. Work is underway to deal more effectively with polyploid genetics	Very suitable application, not attempted yet; Maybe challenging for interspecies hybrids	FR (INRA): 1,200 genotypes of *Populus nigra* are being used to develop intraspecific GS calibrations US: 1,250 genotypes are being used to develop GS calibrations
Traditional transgenesis	Efficient introduction of “foreign” traits (possibly from other genus/species) into an elite plant (e.g., a proven parent or a hybrid) that needs a simple trait to be improved; Validate candidate genes from QTL studies	MAS and knowledge of the biology of the trait and source of genes to confer the relevant changes to phenotype. Working transformation protocol	IP issues/cost of regulatory approval/GMO labelling/marketing issues/tricky to use transgenes in out‐breeders because of complexity of transforming and gene flow risks	US: Many programs in United States are creating transgenic plants, using Alamo as a source of transformable genotypes. Many traits of interest. Nothing commercial yet	CN: Changsha: *Bt* gene transformed into *M.sac*. in 2004, *M. sin* transformed with *MINAC2* gene and *M.sac*. transformed with *Cry2Aa* gene, both with a marker gene by *Agrobacterium*. JP: 1 *M.sin*. transgenic for low temperature tolerance with increased expression of fructans (unpublished) NL: Improving protocol for *M.sin* transformation SK: 2 *M.sin* with *Arabidopsis* and *Brachypodium Phytochrome B* genes (Hwang, Cho, et al., 2014; Hwang, Lim, et al., 2014) UK: 2 *M.sin* and 1 *M.flor*. genotypes variously transformed with four cell wall genes, *ipt* and *uidA* genes using two selection systems by biolistics and *Agrobacterium*. Transformed plants are being analysed US: Preliminary work will be taken forward in 2018–2022 in CABBI	UK: Routine transformation not yet possible. Research to overcome recalcitrance ongoing. Currently trying different species and conditions. Poplar transformation used at present US: Frequently attempted with very limited success	US: 600 transgenic lines have been characterized; mostly performed in the aspen hybrids; reproductive sterility, drought tolerance, with Knockdowns
Genome editing CRISPR	Refinement of existing traits in useful parents or promising hybrids by generating targeted mutations in genes known to control the trait of interest. First, a double‐stranded break is made in the DNA which is repaired by natural DNA repair machinery. Leads to frameshift/SNP/or can use a “repair template” or can be used to insert a transgene into a “safe harbour locus”; It could be used to delete repressors (or transcription factors)	Identification, mapping and sequencing of target genes (from DNA sequence); avoiding/screening out of unintended edits	Many regulatory authorities have not decided whether CRISPR and other genome editing technologies are GMOs or not. If GMOs, see comment on stage 10 above. If not, edited crops will be regulated as conventional varieties	CA: Technology is still too new, and switchgrass genome is very complex; Other laboratories are interested, but not yet moving on this US: One programme so far—USDA in Albany	FR: Initiated in 2016 (MISEDIT project) NL: Initiating in 2018 US: Initiating in 2018, in CABBI UK: Not started	Not possible until transformation achieved	US: CRISPR‐Cas9 and Cpf1 have been successfully developed in *Populus* and are highly efficient; Examples for lignin biosynthesis

Note

BFF: Biomass For the Future; *Bt*:* Bacillus thuringiensis*; CABBI: Center for advanced bioenergy and bioproducts innovation; Cpf1: CRISPR from *Prevotella* and *Francisella* 1; CRISPR: clustered regularly interspaced palindromic repeats; CRISPR‐Cas: CRISPR‐associated; Cry2Aa: crystal toxins 2Aa subfamily produced by *Bt*; FS: full‐sib; GBS: genotyping by sequencing; GEBVs: genomic‐estimated breeding values; GMO: genetically modified organism; GS: genomic selection; GWAS: genomewide association study; INRA: French National Institute for Agricultural Research; IP: intellectual property; IPT: isopentenyltransferase; ISSR: inter‐SSR; *M.flor*.: *Miscanthus floridulus*;* M.sac*.: *M. sacchariflorus*;* M.sin.: M*.* sinensis*; MAS: marker‐assisted selection; MISEDIT: miscanthus gene editing for seed‐propagated triploids; NAC: no apical meristem, ATAF1/2, and cup‐shaped cotyledon2‐like; QTL: quantitative trait locus; SNP: single nucleotide polymorphism; SSR: simple sequence repeats; USDA: US Department of Agriculture.

ISO Alpha‐2 letter country codes.

## 2 SWITCHGRASS

Switchgrass is indigenous to the North American prairies. It is grown from seed and harvested annually using technology similar to that used for pastures. Based on collections from thousands of wild prairie remnants, the genetic resources are roughly divided into lowland and upland ecotypes and there are distinct clades within each ecotype which occur along both latitudinal and longitudinal gradients (Evans et al., 2018; Lu et al., 2013; Zhang et al., 2011). Genotype‐by‐environment interactions (G × E) are strong and must be considered in breeding (Casler, 2012; Casler, Mitchell, & Vogel, 2012). Adaptation to environment is regulated principally by responses to day‐length and temperature. There are also strong genotype × environment interactions between the drier western regions and the wetter eastern regions (Casler et al., 2017).

The growing regions of North America are divided into four adaptation zones for switchgrass, each roughly corresponding to two official hardiness zones. The lowland ecotypes are generally late flowering, high yielding and adapted to warmer climates, but have lower drought and cold resistance than upland ecotypes (Casler, 2012; Casler et al., 2012).

In 2015, the US Department of Agriculture (USDA) National Plant Germplasm System, GRIN ( https://www.ars-grin.gov/npgs/), had 181 switchgrass accessions, of which only 96 were available for distribution due to limitations associated with seed multiplication (Casler, Vogel, & Harrison, 2015). There are well over 2,000 additional uncatalogued accessions (Table 1) held by various universities, but the USDA access to these is also constrained by the effort needed in seed multiplication. Switchgrass is a model herbaceous species for conducting scientific research on biomass (Sanderson, Adler, Boateng, Casler, & Sarath, 2006), but little funding is available for the critical prebreeding work that is necessary to link this biological research to commercial breeding. More than a million genotypes from ~2,000 accessions (seed accessions contain many genotypes) have been phenotypically screened in spaced plant nurseries and ten thousand of the most useful have been genotyped with different technologies, depending on the technology available at the time when these were performed. From these characterized genotypes, parents are selected for exploratory pairwise crosses to produce synthetic populations within ecotypes. Switchgrass, like many grasses, is outcrossing due to a strong genetically controlled self‐incompatibly (akin to the S‐Z‐locus system of other grasses; (Martinez‐Reyna & Vogel, 2002)). Thus, the normal breeding approaches used are F_1_ wide crosses and recurrent selection cycles within synthetic populations.

The scale of these programmes varies from small‐scale conventional breeding, based solely on phenotypic selection (e.g., REAP Canada, Montreal, Quebec), to large programmes incorporating modern molecular breeding methods (e.g., USDA‐ARS, Madison, Wisconsin). Early agronomic research and biomass production efforts were focused on the seed‐based multiplication of promising wild accessions from natural prairies. Cultivars Alamo, Kanlow and Cave‐in‐Rock were popular due to high yield and moderate‐to‐wide adaptation. Conventional breeding approaches focussed on biomass production traits and have led to the development of five cultivars particularly suited to biomass production: Cimarron, EG1101, EG1102, EG2101 and Liberty. The first four of these represent the lowland ecotype and were developed either in Oklahoma or Georgia. Liberty is a derivative of lowland × upland hybrids developed in Nebraska following selection for late flowering, the high yield of the lowland ecotype and cold tolerance of the upland ecotype (Vogel et al., 2014). These five cultivars were all approximately 25–30 years in the making, counting from the initiation of these breeding programmes. Many more biomass‐type cultivars are expected within the next few years as these and other breeding programmes mature. The average rate of gain for biomass yield in long‐term switchgrass breeding programmes has been 1%–4% per year, depending on ecotype, population and location of the breeding programme (Casler & Vogel, 2014; Casler et al., 2018). The hybrid derivative Liberty has a biomass yield 43% higher than the better of its two parents (Casler & Vogel, 2014; Vogel et al., 2014). The development of cold‐tolerant and late‐flowering lowland‐ecotype populations for the northern United States has increased biomass yields by 27% (Casler et al., 2018).

Currently, more than 20 recurrent selection populations are being managed in the United States to select parents for improved yield, yield resilience and compositional quality of the biomass. For the agronomic development and upscaling, high seed multiplication rates need to be combined with lower seed dormancy to reduce both crop establishment costs and risks. Expresso is the first cultivar with significantly reduced seed dormancy which is the first step towards development of domesticated populations (Casler et al., 2015). Most phenotypic traits of interest to breeders require a minimum of 2 years to be fully expressed which results in a breeding cycle that is at least two years. More complicated breeding programmes, or traits that require more time to evaluate, can extend the breeding cycle to 4–8 years per generation, for example, progeny testing for biomass yield or field‐based selection for cold tolerance. Breeding for a range of traits with such long cycles calls for the development of molecular methods to reduce timescales and improve breeding efficiency.

Two association panels of switchgrass have been phenotypically and genotypically characterized to identify quantitative trait loci (QTLs) that control important biomass traits. The northern panel consists of 60 populations, approximately 65% from the upland ecotype. The southern panel consists of 48 populations, approximately 65% from the lowland ecotype. Numerous QTLs have been identified within the northern panel to date (Grabowski et al., 2017). Both panels are the subject of additional studies focused on biomass quality, flowering and phenology, and cold tolerance. Additionally, numerous linkage maps have been created by the pairwise crossing of individuals with divergent characteristics, often to generate four‐way crosses that are analysed as pseudo‐F_2_ crosses (Liu, Wu, Wang, & Samuels, 2012; Okada et al., 2010; Serba et al., 2013; Tornqvist et al., 2018). Individual markers and QTLs identified can be used to design marker‐assisted selection (MAS) programmes to accelerate breeding and increase its efficiency. Genomic prediction and selection (GS) holds even more promise with the potential to double or triple the rate of gain for biomass yield and other highly complex quantitative traits of switchgrass (Casler & Ramstein, 2018; Ramstein et al., 2016). The genome of switchgrass has recently been made public through the Joint Genome Institute ( https://phytozome.jgi.doe.gov/).

Transgenic approaches have been heavily relied upon to generate unique genetic variants, principally for traits related to biomass quality (Merrick & Fei, 2015). Switchgrass is highly transformable using either *Agrobacterium*‐mediated transformation or biolistics bombardment, but regeneration of plants is the bottleneck to these systems. Traditionally, plants from the cultivar Alamo were the only regenerable genotypes, but recent efforts have begun to identify more genotypes from different populations that are capable of both transformation and subsequent regeneration (King, Bray, Lafayette, & Parrott, 2014; Li & Qu, 2011; Ogawa et al., 2014; Ogawa, Honda, Kondo, & Hara‐Nishimura, 2016). Cell wall recalcitrance and improved sugar release are the most common targets for modification (Biswal et al., 2018; Fu et al., 2011). Transgenic approaches have the potential to provide traits that cannot be bred using natural genetic variability. However, they will still require about 10–15 years and will cost $70–100 million for cultivar development and deployment (Harfouche, Meilan, & Altman, 2011). In addition, there is commercial uncertainty due to the significant costs and unpredictable timescales and outcomes of the regulatory approval process in the countries targeted for seed sales. As seen in maize, one advantage of transgenic approaches is that they can easily be incorporated into F_1_ hybrid cultivars (Casler, 2012; Casler et al., 2012), but this does not decrease the time required for cultivar development due to field evaluation and seed multiplication requirements.

The potential impacts of unintentional gene flow and establishment of non‐native transgene sequences in native prairie species via cross‐pollination are also major issues for the environmental risk assessment. These limit further the commercialization of varieties made using these technologies. Although there is active research into switchgrass sterility mechanisms to curb unintended pollen‐mediated gene transfer, it is likely that the first transgenic cultivars proposed for release in the United States will be met with considerable opposition due to the potential for pollen flow to remaining wild prairie sites, which account for <1% of the original prairie land area and are highly protected by various governmental and nongovernmental organizations (Casler et al., 2015). Evidence for landscape‐level, pollen‐mediated gene flow from genetically modified *Agrostis* seed multiplication fields (over a mountain range) to pollinate wild relatives (Watrud et al., 2004) confirms the challenge of using transgenic approaches. Looking ahead, genome editing technologies hold considerable promise for creating targeted changes in phenotype (Burris, Dlugosz, Collins, Stewart, & Lenaghan, 2016; Liu et al., 2018), and at least in some jurisdictions, it is likely that cultivars resulting from gene editing will not need the same regulatory approval as GMOs (Jones, 2015a). However in July 2018, the European Court of Justice (ECJ) ruled that cultivars carrying mutations resulting from gene editing should be regulated in the same way as GMOs. The ECJ ruled that such cultivars be distinguished from those arising from untargeted mutation breeding which is exempted from regulation under Directive 2001/18/EC.

## 3 Miscanthus

Miscanthus is indigenous to eastern Asia and Oceania where it is traditionally used for forage, thatching and papermaking (Xi, 2000; Xi & Jezowkski, 2004). In the 1960s, the high biomass potential of a Japanese genotype, introduced to Europe by Danish nurseryman Aksel Olsen in 1935, was first recognized in Denmark (Linde‐Laursen, 1993). Later, this accession was characterized, described and named as “*M*. × *giganteus”* (Greef & Deuter, 1993; Hodkinson & Renvoize, 2001), commonly abbreviated as *M*x*g*. It is a naturally occurring interspecies triploid hybrid between tetraploid *M. sacchariflorus* (2n = 4x) and diploid *M. sinensis* (2n = 2x). Despite its favourable agronomic characteristics and ability to produce high yields in a wide range of environments in Europe (Kalinina et al., 2017), the risks of reliance on it as a single clone have been recognized. Miscanthus, like switchgrass, is outcrossing due to self‐incompatibility (Jiang et al., 2017). Thus, seeded hybrids are an option for commercial breeding. Miscanthus can also be vegetatively propagated by rhizome or in vitro culture, which allows the development of clones. The breeding approaches are usually based on F1 crosses and recurrent selection cycles within the synthetic populations. There are several breeding programmes that target improvement of miscanthus traits including stress resilience, targeted regional adaptation, agronomic “scalability” through cheaper propagation, faster establishment, lower moisture and ash contents and greater usable yield (Clifton‐Brown et al., 2017).

Germplasm collections specifically to support breeding for biomass started in the late 1980s and early 1990s in Denmark, Germany and the United Kingdom (Clifton‐Brown, Schwarz, & Hastings, 2015). These collections have continued with successive expeditions from European and US teams assembling diverse collections from a wide geographic range in eastern Asia, including from China, Japan, South Korea, Russia and Taiwan (Hodkinson, Klaas, Jones, Prickett, & Barth, 2015; Stewart et al., 2009). Three key miscanthus species for biomass production are *M. sinensis*,* M. floridulus* and *M. sacchariflorus*. *M. sinensis* is widely distributed throughout eastern Asia, with an adaptive range from the subtropics to southern Russia (Zhao et al., 2013). This species has small rhizomes and produces many tightly packed shoots forming a “tuft.” *M. floridulus* has a more southerly adaptive range with a rather similar morphology to *M. sinensis*, but grows taller with thicker stems and is evergreen and less cold‐tolerant than the other miscanthus species. *M. sacchariflorus* is the most northern‐adapted species ranging to 50 °N in eastern Russia (Clark et al., 2016). Populations of diploid and tetraploid *M. sacchariflorus* are found in China (Xi, 2000) and South Korea (Yook, 2016), and eastern Russia, but only tetraploids have been found in Japan (Clark, Jin, & Petersen, 2018).

Germplasm has been assembled from multiple collections over the last century, though some early collections are poorly documented. This historical germplasm has been used to initiate breeding programmes largely based on phenotypic and genotypic characterization. As many of the accessions from these collections are “origin unknown,” crucial environmental envelope data are not available. UK‐led expeditions started in 2006 and continued until 2011 with European and Asian partners and have built up a comprehensive collection of 1,500 accessions from 500 sites across Eastern Asia, including China, Japan, South Korea and Taiwan. These collections were guided using spatial climatic data to identify variation in abiotic stress tolerance. Accessions from these recent collections were planted, following quarantine, in multilocation nursery trials at several locations in Europe to examine trait expression in different environments. Based on the resulting phenotypic and molecular marker data, several studies (a) characterized patterns of population genetic structure (Slavov et al., 2014, 2013); (b) evaluated the statistical power of genomewide association studies (GWASs) and identified preliminary marker–trait associations (Slavov et al., 2014, 2013 ); and (c) assessed the potential of genomic prediction (Davey et al., 2017; Slavov et al., 2018b, 2014). Genomic index selection in particular offers the possibility of exploring scenarios for different locations or industrial markets (Slavov et al., 2018a, 2018b).

Separately, US‐led expeditions also collected about 1,500 accessions between 2010 and 2014 (Clark et al., 2014, 2016, 2018, 2015 ). A comprehensive genetic analysis of the population structure has been produced by RADseq for *M. sinensis* (Clark et al., 2015; Van der Weijde, Kamei, et al., 2017) and *M. sacchariflorus* (Clark et al., 2018). Multilocation replicated field trials have also been conducted on these materials in North America and in Asia. GWAS has been conducted for both *M. sinensis* and a subset of *M. sacchariflorus* accessions (Clark et al., 2016). To date, about 75% of these recent US‐led collections are in nursery trials outside the United States. Due to lengthy US quarantine procedures, these are not yet available for breeding in the United States. However, molecular analyses have allowed us to identify and prioritize sets of genotypes that best encompass the genetic variation in each species.

While most *M. sinensis* accessions flower in northern Europe, very few *M. sacchariflorus* accessions flower even in heated glasshouses. For this reason, the European programmes in the United Kingdom, the Netherlands and France have performed mainly *M. sinensis* (intraspecies) hybridizations (Table 4). Selected progeny become the parents of later generations (recurrent selection, as in switchgrass). Seed sets of up to 400 seed per panicle occur in *M. sinensis*. In Aberystwyth and Illinois, significant efforts to induce synchronous flowering in *M. sacchariflorus* and *M. sinensis* have been made because interspecies hybrids have proven higher yield performance and wide adaptability (Kalinina et al., 2017). In interspecies pairwise crosses in glasshouses, breathable bags and/or large crossing tubes or chambers in which two or more whole plants fit are used for pollination control. Seed sets are lower in bags than in the open air because bags restrict pollen movement while increasing temperatures and reducing humidity (Clifton‐Brown, Senior, & Purdy, 2018). About 30% of attempted crosses produced 10 to 60 seeds per bagged panicle. The seed (thousand seed mass ranges from 0.5 to 0.9 g) is threshed from the inflorescences and sown into modular trays to produce plug plants, which are then planted in field nurseries to identify key parental combinations.

A breeding programme of this scale must serve the needs of different environments, accepting the common purpose is to optimize the interception of solar radiation. An ideal hybrid for a given environment combines adaptation to date of emergence with optimization of traits such as height, number of stems per plant, flowering and senescence time to optimize solar interception to produce a high biomass yield with low moisture content at harvest (Robson, Farrar, et al., 2013; Robson, Jensen, et al., 2013). By 2013/2014, conventional breeding in Europe had produced intra‐ and interspecific fertile seeded hybrids. When a cohort (typically about 5) of outstanding crosses have been identified, it is important to work on related upscaling matters in parallel. These are as follows:

- Assessment of the yield and critical traits in selected hybrids using a network of field trials.
- Efficient cloning of the seed parents. While in vitro and macro‐cloning techniques are used, some genotypes are amenable to neither technique.
- High seed production from field crossing trials conducted in locations where flowering in both seed and pollen parents is likely to happen synchronously.
- Scalable and adapted harvesting, threshing and seed processing methods for producing high seed quality.

The results of these parallel activities need to be combined to identify the upscaling pathway for each hybrid; if this cannot be achieved, the hybrid will likely not be commercially viable. The UK‐led programme with partners in Italy and Germany shows that seedbased multiplication rates of 1:2,000 are achievable several interspecific hybrids (Clifton‐Brown et al., 2017). The multiplication rate of *M. sinensis* is higher, probably 1:5,000–10,000. Conventional cloning from rhizome is limited to around 1:20, that is, one ha could provide rhizomes for around 20 ha of new plantation.

Multilocation field testing of wild and novel miscanthus hybrids selected by breeding programmes in the Netherlands and the United Kingdom was performed as part of the project Optimizing Miscanthus Biomass Production (OPTIMISC, 2012–2016). These trials showed that commercial yields and biomass qualities (Kiesel et al., 2017; Van der Weijde et al., 2017a; Van der Weijde, Kiesel, et al., 2017) could be produced in a wide range of climates and soil conditions from the temperate maritime climate of western Wales to the continental climate of eastern Russia and the Ukraine (Kalinina et al., 2017). Extensive environmental measurements of soil and climate, combined with growth monitoring, are being used to understand abiotic stresses (Nunn et al., 2017; Van der Weijde, Huxley, et al., 2017) and develop genotype‐specific scenarios similar to those reported earlier in Hastings et al. (2009). Phenomics experiments on drought tolerance have been conducted on wild and improved germplasm (Malinowska, Donnison, & Robson, 2017; Van der Weijde, Huxley, et al., 2017). Recently produced interspecific hybrids displaying exceptional yield under drought (~30% greater than control *Mxg*) in field trials in Poland and Moldova are being further studied in detail in the phenomics and genomics facility at Aberystwyth to better understand gene–trait associations which can be fed back into breeding.

Intraspecific seeded hybrids of *M. sinensis* produced in the Netherlands and interspecific *M. sacchariflorus × M. sinensis* hybrids produced by the UK‐led breeding programme have entered yield testing in 2018 with the recently EU‐funded project “GRowing Advanced industrial Crops on marginal lands for biorEfineries (GRACE)” ( https://www.grace-bbi.eu/). Substantial variation in biomass quality for saccharification efficiency (glucose release as % of dry matter), ash content and melting point has already been generated in intraspecific *M. sinensis* hybrids (Van der Weijde, Kiesel, et al., 2017) across environments (Weijde, Dolstra, et al., 2017a). GRACE aims to establish more than 20 hectares of new inter‐ and intraspecific seeded hybrids across six European countries. This project is building the know‐how and agronomy needed to transition from small research plots to commercial‐scale field sites and linking biomass production directly to industrial applications. The biomass produced by hybrids in different locations will be supplied to innovative industrial end‐users making a wide range of biobased products, both for chemicals and for energy. In the United States, multi‐location yield were initiated in 2018 to evaluate new triploid *M*. × *giganteus* genotypes developed at Illinois. Currently, infertile hybrids are favoured in the United States because this eliminates the risk of invasiveness from naturally dispersed, viable seed. The precautionary principle is applied as fertile miscanthus has naturalized in several states (Quinn, Allen, & Stewart, 2010). North European multilocation field trials, in the EMI and OPTIMISC projects, have shown there is minimal risk of invasiveness even in years when fertile flowering hybrids produce viable seed. Naturalized stands have not established here due perhaps to low dormancy, poor overwintering and low seedling competitive strength. In addition to breeding for nonshattering or sterile seeded hybrids, Quinn et al. (2010) suggest management strategies which can further minimize environmental opportunities to manage the risk of invasiveness.

### 3.1 Molecular breeding and biotechnology

In miscanthus, new plant breeding techniques (Table 5) have focussed on developing molecular markers for breeding in Europe, the United States, South Korea and Japan. There are several publications on QTL mapping populations for key traits such as flowering (Atienza, Ramirez, & Martin, 2003) and compositional traits (Atienza, Satovic, Petersen, Dolstra, & Martin, 2003). In the United States and United Kingdom, independent and interconnected bi‐parental “mapping” families have been studied (Dong et al., 2018; Gifford, Chae, Swaminathan, Moose, & Juvik, 2015) alongside panels of diverse germplasm accessions for GWAS (Slavov et al., 2014). Further developments calibrating GS with very large panels of parents and cross progeny are underway (Davey et al., 2017). The recently completed first miscanthus reference genome sequence is expected to improve the efficiency of MAS strategies, and especially GWAS ( https://phytozome.jgi.doe.gov/pz/portal.html#!info?alias=Org_Msinensis_er). For example, without a reference genome sequence, Clark et al. (2014) obtained 21,207 RADseq SNPs (single nucleotide polymorphisms) on a panel of 767 miscanthus genotypes (mostly *M. sinensis*), but subsequent reanalysis of the RADseq data using the new reference genome resulted in hundreds of thousands of SNPs being called.

Robust and effective in vitro regeneration systems have been developed for *Miscanthus sinensis*,* M. × giganteus* and *M. sacchariflorus* (Dalton, 2013; Guo et al., 2013; Hwang, Cho, et al., 2014; Rambaud et al., 2013; Ślusarkiewicz‐Jarzina et al., 2017; Wang et al., 2011; Zhang et al., 2012). However, there is still significant genotype specificity and these methods need “in‐house” optimization and development to be used routinely. They provide potential routes for rapid clonal propagation and also as a basis for genetic transformation. Stable transformation using both biolistics (Wang et al., 2011) and *Agrobacterium tumefaciens* DNA delivery methods (Hwang, Cho, et al., 2014; Hwang, Lim, et al., 2014) has been achieved in *M. sinensis*. The development of miscanthus transformation and gene editing to generate diplogametes for producing seed‐propagated triploid hybrids are performed as part of the French project MISEDIT (miscanthus gene editing for seed‐propagated triploids). There are no reports of genome editing in any miscanthus species, but new breeding innovations, including genome editing, are particularly relevant in this slow‐to‐breed, nonfood, bioenergy crop (Table 4).

## 4 WILLOW

Willow (*Salix* spp.) is a very diverse group of catkin‐bearing trees and shrubs. Willow belongs to the family *Salicaceae*, which also includes the *Populus* genus. There are approximately 350 willow species (Argus, 2007), found mostly in temperate and arctic zones in the northern hemisphere. A few are adapted to subtropical and tropical zones. The centre of diversity is believed to be in Asia, with over 200 species in China. Around 120 species are found in the former Soviet Union, over 100 in North America and around 65 species in Europe, and one species is native to South America (Karp et al., 2011). Willows are dioecious, thus obligate outcrossers, and highly heterozygous. The haploid chromosome number is 19 (Hanley & Karp, 2014). Around 40% of species are polyploid (Suda & Argus, 1968), ranging from triploids to the atypical dodecaploid *S. maxxaliana* with 2n=190 (Zsuffa et al., 1984).

Although almost exclusively native to the Northern Hemisphere, willow has been grown around the globe for many thousands of years to support a wide range of applications (Kuzovkina & Quigley, 2005; Stott, 1992). However, it has been the focus of domestication for bioenergy purposes for only a relatively short period, since the 1970s in North America and Europe. For bioenergy, breeders have focused their efforts on the shrub willows (subgenus *Vetix*) because of their rapid juvenile growth rates as a response to coppicing on a 2‐ to 4‐year cycle that can be accomplished using farm machinery rather than forestry equipment (Shield, Macalpine, Hanley, & Karp, 2015; Smart & Cameron, 2012).

Since shrub willow was not generally recognized as an agricultural crop until very recently, there has been little commitment to building and maintaining germplasm repositories of willow to support long‐term breeding. One exception is the United Kingdom, where a large and well‐characterized *Salix* germplasm collection comprising over 1,500 accessions is held at Rothamsted Research (Stott, 1992; Trybush et al., 2008). Originally initiated for use in basketry in 1923, accessions have been added ever since. In the United States, a germplasm collection of >350 accessions is located at Cornell University to support the breeding programme there. The UK and Cornell collections have a relatively small number of accessions in common (around 20). Taken together, they represent much of the species diversity, but only a small fraction of the overall genetic diversity within the genus. There are three active willow breeding programmes in Europe: Rothamsted Research (UK), Salixenergi Europa AB (SEE) and a programme at the University of Warmia and Mazury in Olsztyn (Poland) (abbreviations used in Table 1). There is one active US programme based at Cornell University. Cultivars are still being marketed by the European Willow Breeding Programme (EWBP) (UK), which was actively breeding biomass varieties from 1996 to 2002. Cultivars are protected by plant breeders’ rights (PBRs) in Europe and by plant patents in the United States. The sharing of genetic resources in the willow community is generally regulated by material transfer agreements (MTA) and tailored licensing agreements, although the import of cuttings into North America is prohibited except under special quarantine permit conditions.

Efforts to augment breeding germplasm collection from nature are continuing, with phenotypic screening of wild germplasm performed in field experiments with 177 *S. purpurea* genotypes in the United States (at sites in Geneva and Portland, NY and Morgantown, WV) that have been genotyped using genotyping by sequencing (GBS) (Elshire et al., 2011). In addition, there are approximately 400 accessions of *S. viminalis* in Europe (near Pustnäs, Uppsala, Sweden and Woburn, UK (Berlin et al., 2014; Hallingbäck et al., 2016). The *S. viminalis* accessions were initially genotyped using 38 simple sequence repeats (SSR) markers to assess genetic diversity and screened with ~1,600 SNPs in genes of potential interest for phenology and biomass traits. Genetics and genomics, combined with extensive phenotyping, have substantially improved the genetic basis of biomass‐related traits in willow and are now being developed in targeted breeding via MAS. This underpinning work has been conducted on large specifically developed biparental *Salix* mapping populations (Hanley & Karp, 2014; Zhou et al., 2018), as well as GWAS panels (Hallingbäck et al., 2016).

Once promising parental combinations are identified, crosses are usually performed using fresh pollen from material that has been subject to a phased removal from cold storage (−4°C) (Lindegaard & Barker, 1997; Macalpine, Shield, Trybush, Hayes, & Karp, 2008; Mosseler, 1990). Pollen storage is useful in certain interspecific combinations where flowering is not naturally synchronized. This can be overcome by using pollen collection and storage protocol which involves extracting pollen using toluene (Kopp, Maynard, Niella, Smart, & Abrahamson, 2002).

The main breeding approach to improve willow yields relies on species hybridization to capture hybrid vigour (Fabio et al., 2017; Serapiglia, Gouker, & Smart, 2014). In the absence of genotypic models for heterosis, breeders have extensively tested general and specific combining ability of parents to produce superior progeny. The UK breeding programmes (EWBP 1996–2002 and Rothamsted Research from 2003 on) have performed more than 1,500 exploratory cross‐pollinations. The Cornell programme has successfully completed about 550 crosses since 1998. Investment into the characterization of genetic diversity combined with progeny tests from exploratory crosses has been used to produce hundreds of targeted intraspecies crosses in the United Kingdom and United States, respectively (see Table 1). To achieve long‐term gains beyond F_1_ hybrids, four intraspecific recurrent selection populations have been created in the United Kingdom (for *S. dasyclados, S. viminalis* and *S. miyabeana*) and Cornell is pursuing recurrent selection of *S. purpurea*. Interspecific hybridizations with genotypes selected from the recurrent selection cycles are well advanced in willow, with such crosses to date totalling 420 in the United Kingdom and over 100 in the United States.

While species hybridization is common in *Salix*, it is not universal. Of the crosses attempted, about 50% hybridize and produce seed (Macalpine, Shield, & Karp, 2010). As the viability of seed from successful crosses is short (a matter of days at ambient temperatures), proper seed rearing and storage protocols are essential (Maroder, Prego, Facciuto, & Maldonado, 2000).

Progeny from crosses are treated in different ways among the breeding programmes at the seedling stage. In the United States, seedlings are planted into an irrigated field where plants are screened for two seasons before being progressed to further field trials. In the United Kingdom, seedlings are planted into trays of compost where they remain containerized in an irrigated nursery for the remainder of year one. In the United Kingdom, seedlings are subject to two rounds of selection in the nursery year. The first round takes place in September to select against susceptibility to rust infection (*Melampsora* spp.). A second round of selection in winter assesses tip damage from frost and giant willow aphid infestation. In the United States where the rust pressure is lower, screening for *Melampsora* spp. cannot be performed at the nursery stage. Both programmes monitor *Melampsora* spp., pest susceptibility, yield and architecture over multiple years in field trials. Selected material is subject to two rounds of field trials followed by a final multilocation yield trial to identify varieties for commercialization.

Promising selections (i.e., potential cultivars) need to be clonally propagated. A rapid, in vitro tissue culture propagation method has been developed (Palomo‐Ríos et al., 2015). This method can generate about 5,000 viable, transplantable clones from a single plant in just 24 weeks. An in vitro system can also accommodate early selection via molecular or biochemical markers to increase selection speed. Conventional breeding systems take 13 years via four rounds of selection from crossing to selecting a variety (Figure 1), but this has the potential to be reduced to 7 years if micropropagation and MAS selection are adopted (Hanley & Karp, 2014; Palomo‐Ríos et al., 2015).

Willows are currently propagated commercially by planting winter‐dormant stem cuttings in spring. Commercial planting systems for willow use mechanical planters that cut and insert stem sections from whips into a well‐prepared soil. One hectare of stock plants grown in specific multiplication beds planted at 40,000 plants per ha produces planting material for 80 hectares of commercial short‐rotation coppice willow annually (planted at 15,000 cuttings per hectare) (Whittaker et al., 2016). When commercial plantations are established, the industry standard is to plant intimate mixtures of ~5 diverse rust (*Melampsora* spp.)‐resistant varieties (McCracken & Dawson, 1997; Van Den Broek et al., 2001).

The foundations for using new plant breeding techniques have been established with funding from both the public and the private sectors. To establish QTL maps, 16 mapping populations from biparental crosses are under study in the United Kingdom. Nine are under study in the United States. The average number of individuals in these families ranges from 150 to 947 (Hanley & Karp, 2014). GS is also being evaluated in *S. viminalis*, and preliminary results indicate that multiomic approaches combining genomic and metabolomic data have great potential (Slavov & Davey, 2017). For both QTL and GS approaches, the field phenotyping demands are large as several thousand individuals need to be phenotyped for a wide range of traits. These include the following: dates of bud burst and growth succession, stem height, stem density, wood density and disease resistance. The greater the number of individuals, the more precise the QTL marker maps and GS models are. However, the logistical and financial challenges of phenotyping large numbers of individuals are considerable, because the willow crop is >5 m tall in the second year. There is tremendous potential to improve the throughput of phenotyping using unmanned aerial systems, which is being tested in the USDA National Institute of Food and Agriculture (NIFA) Willow SkyCAP project at Cornell. Further, investment in these approaches needs to be sustained over many years fully realizes the potential of a marker‐assisted selection programme for willow.

To date, despite considerable efforts in Europe and the United States to establish a routine transformation system, there has not been a breakthrough in willow, but attempts are ongoing. As some form of transformation is typically a prerequisite for genome editing techniques, these have not yet been applied to willow.

In Europe, there are 53 short‐rotation coppice (SRC) biomass willow cultivars registered with the Community Plant Variety Office (CPVO) for PBRs, of which ~25 are available commercially in the United Kingdom. There are eight patented cultivars commercially available in the United States. In Sweden, there are nine commercial cultivars registered in Europe and two others which are unregistered ( https://salixenergi.se/planting-material/). Furthermore, there are about 20 precommercial hybrids in final yield trials in both the United States and the United Kingdom. It has been estimated that it would take two years to produce the stock required to plant 50 ha commercially from the plant stock in the final yield trials. Breeding programmes have already delivered rust‐resistant varieties and increases in yield to the market. The adoption of advanced breeding technologies will likely lead to a step change in improving traits of interest.

## 5 POPLAR

Poplar, a fast‐growing tree from the northern hemisphere with a small genome size, has been adopted for commercial forestry and scientific purposes. The genus *Populus* consists of about 29 species, classified in six different sections: *Populus* (formerly *Leuce*), *Tacamahaca*,* Aigeiros*,* Abaso*,* Turanga* and *Leucoides* (Eckenwalder, 1996). The *Populus* species of most interest for breeding and testing in the United States and Europe are *P. nigra, P. deltoides*,* P. maximowiczii* and *P. trichocarpa* (Stanton, 2014). *Populus* clones for biomass production are being developed by intra‐ and interspecies hybridization (DeWoody, Trewin, & Taylor, 2015; Richardson, Isebrands, & Ball, 2014; van der Schoot et al., 2000). Recurrent selection approaches are used for gradual population improvement and to create elite clonal lines for commercialization (Berguson, McMahon, & Riemenschneider, 2017; Neale & Kremer, 2011). Currently, poplar breeding in the United States occurs in industrial and academic programmes located in the Southeast, the Midwest and the Pacific Northwest. These use six species and five interspecific taxa (Stanton, 2014).

The southeastern programme historically focused on recurrent selection of *P. deltoides* from accessions made in the lower Mississippi River alluvial plain (Robison, Rousseau, & Zhang, 2006). More recently, the genetic base has been broadened to produce interspecific hybrids with resistance to the fungal infection *Septoria musiva,* which causes cankers.

In the midwest of the United States, population improvement efforts are focused on *P. deltoides* selections from native provenances and hybrid crosses with accessions introduced from Europe. Interspecific, intercontinental (Europe and America) hybrid crosses between *P. nigra* and *P. deltoides* (*P. × canadensis*) are behind many of the leading commercial hybrids which are the most advanced breeding materials for many applications and regions. In Minnesota, previous breeding experience and efforts utilizing *P. maximowiczii* and *P. trichocarpa* have been discontinued due to *Septoria* susceptibility and a lack of cold hardiness (Berguson et al., 2017). Traits targeted for improvement include yield/growth rate, cold hardiness, adventitious rooting, resistance to *Septoria* and *Melampsora* leaf rust, and stem form. The Upper Midwest programme also carries out wide hybridizations within the section Populus. The *P. × wettsteinii (P. tremula × P. tremuloides)* taxon is bred for gains in growth rate, wood quality and resistance to the fungus *Entoleuca mammata* which causes hypoxylon canker (David & Anderson, 2002).

In the Pacific Northwest, GreenWood Resources Inc. leads poplar breeding that emphasizes interspecific hybrid improvement of *P. × generosa* (*P. deltoides* *× P. trichocarpa* and reciprocal) and *P. deltoides × P. maximowiczii* taxa for coastal regions, and the *P. × canadensis* taxon for the drier, continental regions. Intraspecific improvement of second‐generation breeding populations of *P. deltoides, P. nigra, P. maximowiczii* and *P. trichocarpa* are also involved (Stanton et al., 2010). The present focus of GreenWood Resources’ hybridization is bioenergy feedstock improvement concentrating on coppice yield, wood‐specific gravity and rate of sugar release.

Industrial interest in poplar in the United States has historically come from the pulp and paper sector, although veneer and dimensional lumber markets have been pursued at times. Currently, the biomass market for liquid transportation fuels is being emphasized, along with the use of traditional and improved poplar genotypes for ecosystem services such as phytoremediation (Tuskan & Walsh, 2001; Zalesny et al., 2016).

In Europe, there are breeding programmes in France, Germany, Italy and Sweden. These include the following: (a) Alasia Franco Vivai (AFV) programme in northern Italy; (b) the French programme led by the poplar Scientific Interest Group (GIS Peuplier) and carried out collaboratively between the National Institute for Agricultural Research (INRA), the National Research Unit of Science and Technology for Environment and Agriculture (IRSTEA) and the Forest, Cellulose, Wood, Construction and Furniture Technology Institute (FCBA); (c) the German programme at Northwest German Forest Research Station (NW‐FVA) at Hannoversch Münden; and (d) the Swedish programme at the Swedish University of Agricultural Sciences and SweTree Technologies AB (Table 1).

AFV leads an Italian poplar breeding programme using extensive field‐grown germplasm collections of *P. alba*,* P. deltoides, P. nigra* and *P. trichocarpa*. While interspecific hybridization uses several taxa, the focus is on *P. × canadensis*. The breeding programme addresses disease resistance (*Marssonina brunnea, Melampsora larici‐populina, Discosporium populeum* and poplar mosaic virus), growth rate and photoperiod adaptation. AFV and GreenWood Resources collaborate in poplar improvement in Europe through the exchange of frozen pollen and seed for reciprocal breeding projects. Plantations in Poland and Romania are currently the focus of the collaboration.

The ongoing French GIS Peuplier is developing a long‐term breeding programme based on intraspecific recurrent selection for the four parental species (*P. deltoides*,* P. trichocarpa*,* P. nigra* and *P. maximowiczii*) designed to better benefit from hybrid vigour demonstrated by the interspecific crosses *P. canadensis, P. deltoides × P. trichocarpa* and *P. trichocarpa × P. maximowiczii*. Current selection priorities are targeting adaptation to soil and climate conditions, resistance and tolerance to the most economically important diseases and pests, high volume production under SRC and traditional poplar cultivation regimes as well as wood quality of interest by different markets. Currently, genomic selection is under exploration to increase selection accuracy and selection intensity while maintaining genetic diversity over generations.

The German NW‐FVA programme is breeding intersectional Aigeiros–Tacamahaca hybrids with a focus on resistance to *Pollaccia elegans, Xanthomonas populi, Dothichiza* spp.*, Marssonina brunnea* and *Melampsora* spp. (Stanton, 2014). Various cross combinations of *P. maximowiczii*,* P. trichocarpa, P. nigra* and *P. deltoides* have led to new cultivars suitable for deployment in varietal mixtures of five to ten genotypes of complementary stature, high productivity and phenotypic stability (Weisgerber, 1993). The current priority is the selection of cultivars for high‐yield, short‐rotation biomass production. Six hundred *P. nigra* genotypes are maintained in an ex situ conservation programme. An in situ* P. nigra* conservation effort involves an inventory of native stands which have been molecular fingerprinted for identity and diversity.

The Swedish programme is concentrating on locally adapted genotypes used for short‐rotation forestry (SRF) because these meet the needs of the current pulping markets. Several field trials have shown that commercial poplar clones tested and deployed in Southern and Central Europe are not well adapted to photoperiods and low temperatures in Sweden and in the Baltics. Consequently, Swedish University of Agricultural Sciences and SweTree Technologies AB started breeding in Sweden in 1990s to produce poplar clones better adapted to local climates and markets.

### 5.1 Molecular breeding technologies

Poplar genetic improvement cannot be rapidly achieved through traditional methods alone because of the long breeding cycles, outcrossing breeding systems and high heterozygosity. Integrating modern genetic, genomic and phenomics techniques with conventional breeding has the potential to expedite poplar improvement.

The genome of poplar has been sequenced (Tuskan et al., 2006). It has an estimated genome size of 485 ± 10 Mbp divided into 19 chromosomes. This is smaller than other PBCs and makes poplar more amenable to genetic engineering (transgenesis), GS and genome editing. Poplar has seen major investment in both the United States and Europe, being the model system for woody perennial plant genetics and genomics research.

### 5.2 Targets for genetic modification

Traits targeted include wood properties (lignin content and composition), early/late flowering, male sterility to address biosafety regulation issues, enhanced yield traits and herbicide tolerance. These extensive transgenic experiments have shown differences in recalcitrance to in vitro regeneration and genetic transformation in some of the most important commercial hybrid poplars (Alburquerque et al., 2016). Further, transgene expression stability is being studied. So far, China is the only country known to have commercially used transgenic, insect‐resistant poplar. A precommercial herbicide‐tolerant poplar was trialled for 8 years in the United States (Li, Meilan, Ma, Barish, & Strauss, 2008) but could not be released due to stringent environmental risk assessments required for regulatory approval. This increases translation costs and delays reducing investor confidence for commercial deployment (Harfouche et al., 2011).

The first field trials of transgenic poplar were performed in France in 1987 (Fillatti, Sellmer, Mccown, Haissig, & Comai, 1987) and in Belgium in 1988 (Deblock, 1990). Although there have been a total of 28 research‐scale GM poplar field trials approved in the European Union under Council Directive 90/220/EEC since October 1991 (in Poland, Belgium, Finland, France, Germany, Spain, Sweden and in the United Kingdom (Pilate et al., 2016), only authorizations in Poland and Belgium are in place today. In the United States, regulatory notifications and permits for nearly 20,000 transgenic poplar trees derived from approximately 600 different constructs have been issued since 1995 by the USDA's Animal and Plant Health Inspection Service (APHIS) (Strauss et al., 2016).

### 5.3 Genome editing CRISPR technologies

Clustered regularly interspaced palindromic repeats (CRISPR) and the CRISPR‐associated (CRISPR‐Cas) nucleases are a groundbreaking genome‐engineering tool that complements classical plant breeding and transgenic methods (Moreno‐Mateos et al., 2017). Only two published studies in poplar have applied the CRISPR/Cas9 technology. One is in *P. tomentosa*, in which an endogenous phytoene desaturase gene (*PtoPDS*) was successfully disrupted site specifically in the first generation of transgenic plants resulting in an albino and dwarf phenotype (Fan et al., 2015). The second was in *P. tremula × alba*, in which high CRISPR‐Cas9 mutational efficiency was achieved for three 4‐coumarate:CoA ligase (4Cl) genes, 4CL1, 4CL2 and 4CL5, associated with lignin and flavonoid biosynthesis (Zhou et al., 2015). Due to its low cost, precision and rapidness, it is very probable that cultivars or clones produced using CRISPR technology will be ready for marketing in the near future (Yin et al., 2017). Recently, a CRISPR with a smaller associated endonuclease has been discovered from *Prevotella* and *Francisella* 1 (Cpf1) which may have advantages over Cas9. In addition, there are reports of DNA‐free editing in plants, using both CRISPR Cpf1 and CRISPR Cas9, for example, Ref (Kim et al., 2017; Mahfouz, 2017; Zaidi et al., 2017).

It remains unresolved whether plants modified by genome editing will be regulated as genetically modified organisms (GMOs) by the relevant authorities in different countries (Lozano‐Juste & Cutler, 2014). Regulations to cover these new breeding techniques are still evolving, but those countries who have published specific guidance (including United States, Argentina and Chile) are indicating that plants possessing simple genome edits will not be regulated as conventional transgenesis (Jones, 2015b). The first generation of genome‐edited crops will likely be phenocopy gene knockouts that already exist to produce “nature identical” traits, that is, traits that could also be derived by conventional breeding. Despite this, confidence in applying these new powerful breeding tools remains limited owing to the uncertain regulatory environment in many parts of the world (Gao, 2018) including the recent ECJ 2018 rulings mentioned earlier.

### 5.4 Genomics‐based breeding technologies

Poplar breeding programs are becoming well equipped with useful genomics tools and resources that are critical to explore genomewide variability and make use of the variation for enhancing genetic gains. Deep transcriptome sequencing, resequencing of alternate genomes and GBS technology for genomewide marker detection using next‐generation sequencing (NGS) are yielding valuable genomics tools. GWAS with NGS‐based markers facilitates marker identification for MAS, breeding by design and GS.

GWAS approaches have provided a deeper understanding of genome function as well as allelic architectures of complex traits (Huang et al., 2010) and have been widely implemented in poplar for wood characteristics (Porth et al., 2013), stomatal patterning, carbon gain versus disease resistance (McKown et al., 2014), height and phenology (Evans et al., 2014), cell wall chemistry (Muchero et al., 2015), growth and cell walls traits (Fahrenkrog et al.., 2017), bark roughness (Bdeir et al., 2017) and height and diameter growth (Liu et al., 2018). Using high‐throughput sequencing and genotyping platforms, an enormous amount of SNP markers have been used to characterize the linkage disequilibrium (LD) in poplar (e.g., Slavov et al., 2012, discussed below).

The genetic architecture of photoperiodic traits in perennial trees is complex involving many loci. However, it shows high levels of conservation during evolution (Maurya & Bhalerao, 2017). These genomics tools can therefore be used to address adaptation issues and fine‐tune the movement of elite lines into new environments. For example, poor timing of spring bud burst and autumn bud set can result in frost damage resulting in yield losses (Ilstedt, 1996). These have been studied in *P. tremula* genotypes along a latitudinal cline in Sweden (~56–66^o^N) and have revealed high nucleotide polymorphism in two nonsynonymous SNPs within and around the *phytochrome B2* locus (Ingvarsson, Garcia, Hall, Luquez, & Jansson, 2006; Ingvarsson, Garcia, Luquez, Hall, & Jansson, 2008). Resequencing 94 of these *P. tremula* genotypes for GWAS showed that noncoding variation of a single genomic region containing the *PtFT2* gene described 65% of observed genetic variation in bud set along the latitudinal cline (Tan, 2018).

Resequencing genomes is currently the most rapid and effective method detecting genetic differences between variants and for linking loci to complex and important agronomical and biomass traits, thus addressing breeding challenges associated with long‐lived plants like poplars.

To date, whole genome resequencing initiatives have been launched for several poplar species and genotypes. In *Populus*, LD studies based on genome resequencing suggested the feasibility of GWAS in undomesticated populations (Slavov et al., 2012). This plant population is being used to inform breeding for bioenergy development. For example, the detection of reliable phenotype/genotype associations and molecular signatures of selection requires a detailed knowledge about genomewide patterns of allele frequency variation, LD and recombination, suggesting that GWAS and GS in undomesticated populations may be more feasible in *Populus* than previously assumed. Slavov et al. (2012) have resequenced 16 genomes of *P. trichocarpa* and genotyped 120 trees from 10 subpopulations using 29,213 SNPs (Geraldes et al., 2013). The largest ever SNP data set of genetic variations in poplar has recently been released, providing useful information for breeding https://www.bioenergycenter.org/besc/gwas/index.cfm. Also, deep sequencing of transcriptomes using RNA‐Seq has been used for identification of functional genes and molecular markers, that is, polymorphism markers and SSRs. A multitissue and multiple experimental data set for *P. trichocarpa* RNA‐Seq is publicly available ( https://jgi.doe.gov/doe-jgi-plant-flagship-gene-atlas/).

The availability of genomic information of DNA‐containing cell organelles (nucleus, chloroplast and mitochondria) will also allow a holistic approach in poplar molecular breeding in the near future (Kersten et al., 2016). Complete *Populus* genome sequences are available for nucleus (*P. trichocarpa*; section Tacamahaca) and chloroplasts (seven species, and two clones from *P. tremula* W52 and *P. tremula* *× P. alba* 717–1B4). A comparative approach revealed structural and functional information, broadening the knowledge base of *Populus* cpDNA and stimulating future diagnostic marker development. The availability of whole genome sequences of these cellular compartments of *P. tremula* holds promise for boosting marker‐assisted poplar breeding. Other nuclear genome sequences from additional *Populus* species are now available (e.g., *P. deltoides* ( https://phytozome.jgi.doe.gov/pz/) and will become available in the forthcoming years (e.g., *P. tremula* and *P. tremuloides*—PopGenIE (Sjodin, Street, Sandberg, Gustafsson, & Jansson, 2009)). Recently, the characterization of the poplar pan‐genome by genomewide identification of structural variation in three crossable poplar species, *P. nigra*,* P. deltoides* and *P. trichocarpa,* revealed a deeper understanding of the role of inter‐ and intraspecific structural variants in poplar phenotype and may have important implications for breeding, particularly, interspecific hybrids (Pinosio *et al.,* 2016).

GS has been proposed as an alternative to MAS in crop improvement (Bernardo & Yu, 2007; Heffner, Sorrells, & Jannink, 2009). GS is particularly well suited for species with long generation times, for characteristics that display moderate‐to‐low heritability, for traits that are expensive to measure and for selection of traits expressed late in the life cycle, as is the case for most traits of commercial value in forestry (Harfouche et al., 2012). Current joint genome sequencing efforts to implement GS in poplar using genomic‐estimated breeding values for bioenergy conversion traits from 49 *P. trichocarpa* families and 20 full‐sib progeny are taking place at the Oak Ridge National Laboratory and GreenWood Resources (Brian Stanton, personal communication https://cbi.ornl.gov/). These data together with the resequenced GWAS population data will be the basis for developing GS algorithms. Genomic breeding tools have been developed for the intraspecific programme targeting yield, resistance to *Venturia* shoot blight, *Melampsora* leaf rust, resistance to *Cryptorhynchus lapathi*, stem form, wood‐specific gravity and wind firmness (Evans et al., 2014; Guerra et al., 2016). A newly developed “breeding with rare defective alleles” (BRDA) technology has been developed to exploit natural variation of *P. nigra* and identify defective variants of genes predicted by prior transgenic research to impact lignin properties. Individual trees carrying naturally defective alleles can then be incorporated directly into breeding programs, thereby bypassing the need for transgenics (Vanholme et al., 2013). This novel breeding technology offers a reverse genetics complement to emerging GS for targeted improvement of quantitative traits (Tsai, 2013).

### 5.5 Phenomics‐assisted breeding technology

Phenomics involves the characterization of phenomes—the full set of phenotypes of given individual plants (Houle, Govindaraju, & Omholt, 2010). Traditional phenotyping tools, which inefficiently measure a limited set of phenotypes, have become a bottleneck in plant breeding studies. High‐throughput plant phenotyping facilities provide accurate screening of thousands of plant breeding lines, clones or populations over time (Fu, 2015) are critical for accelerating genomics‐based breeding. Automated image collection and analysis, phenomics technologies allow accurate and nondestructive measurements of a diversity of phenotypic traits in large breeding populations (Gegas, Gay, Camargo & Doonan, 2014; Goggin, Lorence, & Topp, 2015; Ludovisi et al., 2017; Shakoor, Lee, & Mockler, 2017). One important consideration is the identification of relevant and quantifiable target traits that are early diagnostic indicators of biomass yield. Good progress has been made in elucidating these underpinning morpho‐physiological traits that are amenable to remote sensing in *Populus* (Harfouche, Meilan, & Altman, 2014; Rae, Robinson, Street, & Taylor, 2004). More recently, Ludovisi et al. (2017) developed a novel methodology for field phenomics of drought stress in a *P. nigra* F_2_ partially inbred population using thermal infrared images recorded from an unmanned aerial vehicle‐based platform.

Energy is the current main market for poplar biomass, but the market return provided is not sufficient to support production expansion even with added demand for environmental and land management “ecosystem services” such as the treatment of effluent, phytoremediation, riparian buffer zones and agro‐forestry plantings. Aviation fuel is a significant target market (Crawford et al., 2016). To serve this market and to reduce current carbon costs of production (Budsberg et al., 2016), key improvement traits in addition to yield (e.g., coppice regeneration, pest/disease resistance, water‐ and nutrient‐use efficiencies) will be trace greenhouse gas (GHG) emissions (e.g., isoprene volatiles), site adaptability and biomass conversion efficiency. Efforts are underway to have national environmental protection agencies’ approval for poplar hybrids qualifying for renewable energy credits.

## 6 REFLECTIONS ON THE COMMERCIALIZATION CHALLENGE

**Figure 2:**
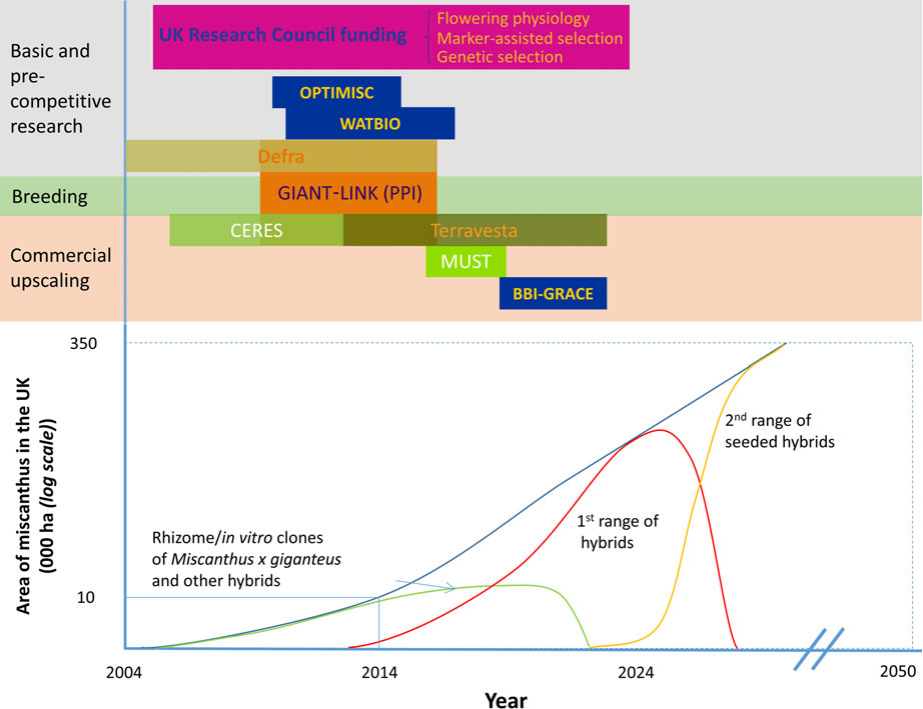
A schematic development pathway for miscanthus in the United Kingdom related to the investment in R&D projects at Aberystwyth (top coloured areas for projects in the three categories: basic research, breeding and commercial upscaling) leading to a projected cropping area of 350,000 ha by 2030 with clonal and successive ranges of improved seed‐based hybrids. Purple represents the Biotechnology and Biological Sciences Research Council (BBSRC) and brown the Department for Environment, Food and Rural Affairs (Defra) (UK National funding); blue bars represent EU funding and green private sector funding (Terravesta and CERES); and GIANT‐LINK and Miscanthus Upscaling Technology (MUST) are public–private‐initiatives (PPI)

The research and innovation activities reviewed in this paper aim to advance the genetic improvement of species that can provide feedstocks for bioenergy applications should those markets eventually develop. These markets need to generate sufficient revenue and adequately distribute it to the actors along the value chain. The work on all four crops shares one thing in common: long‐term efforts to integrate fundamental knowledge into breeding and crop development along a research and development (R&D) pipeline. The development of miscanthus led by Aberystwyth University exemplifies the concerted research effort that has integrated the R&D activities from eight projects over 14 years with background core research funding along an emerging innovation chain (Figure 2). This programme has produced a first range of conventionally bred seeded interspecies hybrids, which are now in upscaling trials (Table 3). The application of molecular approaches (Table 5) with further conventional breeding (Table 4) offers the prospect of a second range of improved seeded hybrids. This example shows that research‐based support of the development of new crops or crop types requires a long‐term commitment that goes beyond that normally available from project‐based funding (Figure 1). Innovation in this sector requires continuous resourcing of conventional breeding operations and capability to minimize time and investment losses caused by funding discontinuities.

**Figure 3:**
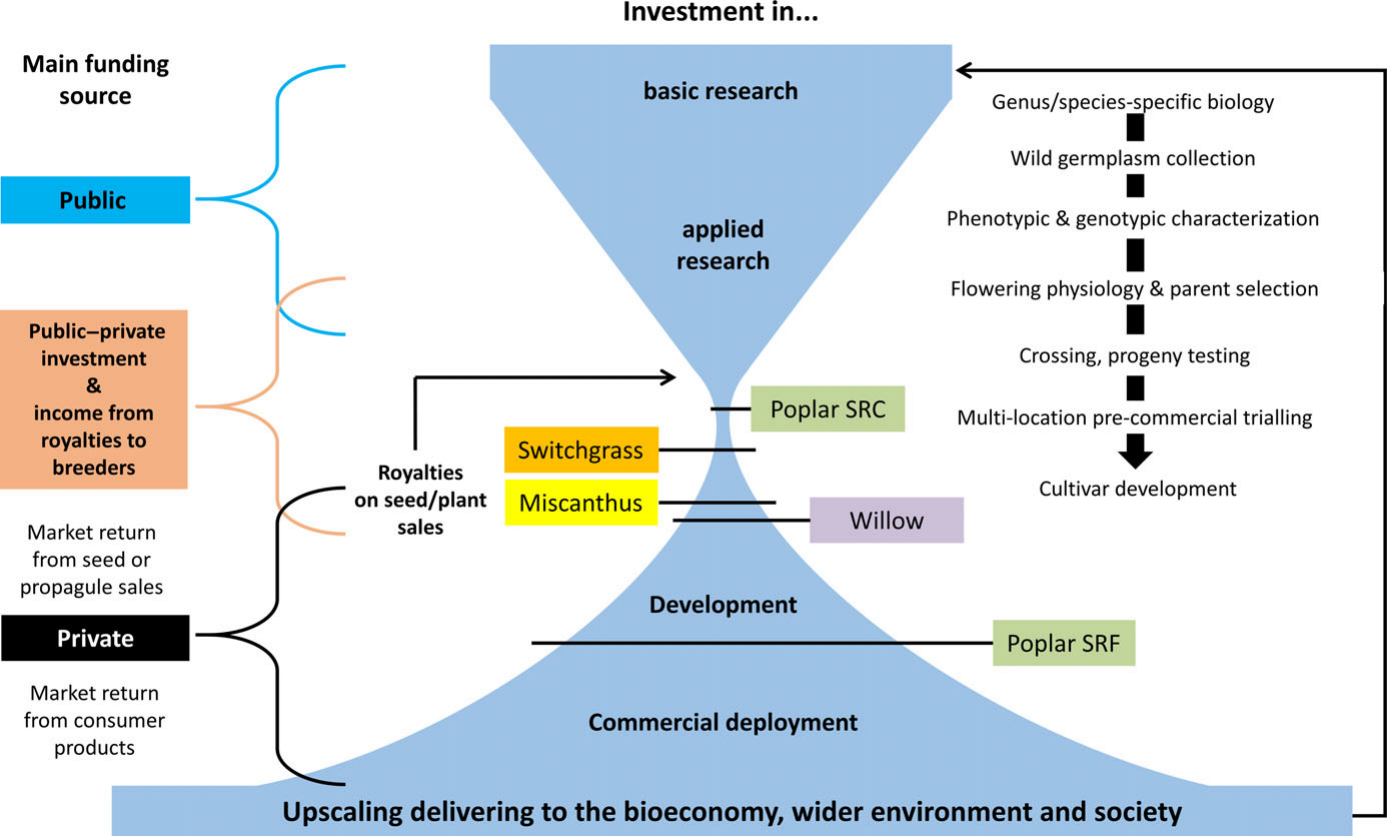
A schematic relating some of the steps in the innovation chain from relatively basic crop science research through to the deployment in commercial cropping systems and value chains. The shape of the funnel above the expanding development and deployment represents the availability of investment along the development chain from relatively basic research at the top to the upscaled deployment at the bottom. Plant breeding links the research effort with the development of cropping systems. The constriction represents the constrained funding for breeding that links conventional public research investment and the potential returns from commercial development. The handover points between publicly funded work to develop the germplasm resources (often known as prebreeding), the breeding and the subsequent crop development are shown on the left. The constriction point is aggravated by the lack academic rewards for this essential breeding activity. The outcome is such that this innovation system is constrained by the precarious resourcing of plant breeding. The authors’ assessment of development status of the four species is shown (poplar having two: one for short‐rotation coppice (SRC) poplar and one for the more traditional short‐rotation forestry (SRF)). The four new perennial biomass crops (PBCs) are now in the critical phase of depending of plant breeding progress without the income stream from a large crop production base

This challenge is increased further by the well‐known market failure in the breeding of many agricultural crop species. The UK Department for Environment, Food and Rural Affairs (Defra) examined the role of genetic improvement in relation to nonmarket outcomes, such as environmental protection, and concluded that public investment in breeding was required if profound market failure is to be addressed (Defra, 2002). With the exception of widely grown hybrid crops, such as maize, and some high‐value horticultural crops, royalties arising from plant breeders’ rights or other returns to breeders fail to adequately compensate for the full cost for research‐based plant breeding. The result, even for major crops such as wheat, is sub‐optimal investment and suboptimal returns for society. This market failure is especially acute for perennial crops developed for improved sustainability, rather than consumer appeal (Tracy et al., 2016). Figure 3 illustrates the underlying challenge of capturing value for the breeding effort. The “valley of death” that results from the low and delayed returns to investment applies generally to the research‐to‐product innovation pipeline (Beard, Ford, Koutsky, & Spiwak, 2009) and certainly to most agricultural crop species. However, this schematic is particularly relevant to PBCs. Most of the value for society from the improved breeding of these crops comes from changes in how agricultural land is used, that is, it depends on the increased production of these crops. The value for society includes many ecosystems benefits: the effects of a return to seminatural perennial crop cover that protects soils, the increase in soil carbon storage, the protection of vulnerable land or the cultivation of polluted soils and the reductions in GHG emissions (Lewandowski, 2016). By its very nature, the production of biomass on agricultural land marginal for food production challenges farm‐level profitability. The costs of planting material and one‐time nature of crop establishment are major early‐stage costs, and therefore, the opportunities for conventional royalty capture by breeders that are manifold for annual crops are limited for PBCs (Hastings *et al.,* 2017). Public investment in developing PBCs for the nonfood biobased sector needs to provide more long‐term support for this critical foundation to a sustainable bioeconomy.

## 7 CONCLUSIONS

This paper provides an overview of research‐based plant breeding in four leading PBCs. For all four PBC genera, significant progress has been made in genetic improvement through collaboration between research scientists and those operating ongoing breeding programmes. Compared with the main food crops, most PBC breeding programmes date back only a few decades (Table 1). This breeding effort has thus co‐evolved with molecular biology and the resulting ‐omics technologies that can support breeding. The development of all four PBCs has depended strongly on public investment in research and innovation. The nature and driver of the investment varied. In close association with public research organizations or universities, all these programmes started with germplasm collection and characterization, which underpin the selection of parents for exploratory wide crosses for progeny testing (Figure 1, Table 4).

Public support for switchgrass in North America was explicitly linked to plant breeding with 12 breeding programmes supported in the United States and Canada. Switchgrass breeding efforts to date, using conventional breeding, have resulted in over 36 registered cultivars in the United States (Table 1), with the development of dedicated biomass‐type cultivars coming within the past few years. While ‐omics technologies have been incorporated into several of these breeding programmes, they have not yet led to commercial deployment in either conventional or hybrid cultivars.

Willow genetic improvement was led by the research community closely linked to plant breeding programmes. Willow and poplar have the longest record of public investment in genetic improvement that can be traced back to 1920s in the United Kingdom and United States, respectively. Like switchgrass, breeding programmes for willow are connected to public research efforts. The United Kingdom, in partnership with the programme based at Cornell University, remains the European leader in willow improvement with a long‐term breeding effort closely linked to supporting biological research at Rothamsted. In willow, F_1_ hybrids have produced impressive yield gains over parental germplasm by capturing hybrid vigour. Over 30 willow clones are commercially available in the United States and Europe, and a further ~90 are under precommercial testing (Table 1).

Compared with willow and poplar, miscanthus is a relative newcomer with all the current breeding programmes starting in the 2000s. Clonal *M. × giganteus* propagated by rhizomes is expected to be replaced by more readily scalable seeded hybrids from intra‐ (*M. sinensis*) and inter‐ (*M. sacchariflorus × M. sinensis*) species crosses with high seed multiplication rates (of >2,000). The first group of hybrid cultivars is expected to be market‐ready around 2022.

Of the four genera used as PBCs, *Populus* is the most advanced in terms of achievements in biological research as a result of its use as a model for basic research of trees. Much of this biological research is not directly connected to plant breeding. Nevertheless, reflecting the fact that poplar is widely grown as a single‐stem tree in SRF, there are about 60 commercially available clones and an additional 80 clones in commercial pipelines (Table 1). Transgenic poplar hybrids have moved beyond proof of concept to commercial reality in China.

Many PBC programmes have initiated long‐term conventional recurrent selection breeding cycles for population improvement, which is a key process in increasing yield through hybrid vigour. As this approach requires many years, most programmes are experimenting with molecular breeding methods as these have the potential to accelerate precision breeding. For all four PBCs, investments in basic genetic and genomic resources, including the development of mapping populations for QTLs and whole genome sequences, are available to support long‐term advances. More recently, association genetics with panels of diverse germplasm are being used as training populations for GS models (Table 5). These efforts are benefitting from publicly available DNA sequences and whole genome assemblies in crop databases. Key to these accelerated breeding technologies are developments in novel phenomics technologies to bridge the genotype/phenotype gap. In poplar, novel remote sensing field phenotyping is now being deployed to assist breeders. These advances are being combined with in vitro and in planta modern molecular breeding techniques such as CRISPR (Table 5). CRISPR technology for genome editing has been proven in poplar. This technology is also being applied in switchgrass and miscanthus (Table 5), but the future of CRISPR in commercial breeding for the European market is uncertain in the light of recent ECJ 2018 rulings.

There is integration of research and plant breeding itself in all four PBCs. Therefore, estimating the ongoing costs of maintaining these breeding programmes is difficult. Investment in research also seeks wider benefits associated with technological advances in plant science rather than cultivar development per se. However, in all cases, the conventional breeding cycle shown in Figure 1 is the basic “engine” with molecular technologies (‐omics) serving to accelerate this engine. The history of the development shows that the existence of these breeding programmes is essential to gain benefits from the biological research. Despite this, it is this essential step that is at most risk from reductions in investment. A conventional breeding programme typically requires a breeder and several technicians who are supported over the long term (20–30 years, Figure 1) at costs of about 0.5 to 1.0 million Euro per year (as of 2018). The analysis reported here shows that the time needed to perform one cycle of conventional breeding, bringing germplasm from the wild to a commercial hybrid ranged from 11 years in switchgrass to 26 years in poplar (Figure 1). In a mature crop grown on over 100,000 ha, with effective cultivar protection and a suitable business model, this level of revenue could come from royalties. Until such levels are reached, PBCs lie in the innovation valley of death (Figure 3) and need public support.

Applying industrial “technology readiness levels” (TRL) originally developed for aerospace (Héder, 2017) to our plant breeding efforts, we estimate many promising hybrids cultivars are at TRL levels of 3–4. In Table 3, experts in each crop estimate that it would take 3 years from now to upscale planting material from leading cultivars in plot trials to 100 ha.

Taking the UK example mentioned in the introduction, planting rates of ~35,000 ha per year from 2022 onwards are needed to reach over 1 m ha by 2050. Ongoing work in the UK‐funded Miscanthus Upscaling Technology (MUST) project shows that ramping annual hybrid seed production from the current level of sufficient seed for 10 ha in 2018 to 35,000 ha would take about 5 years, assuming no setbacks. If current hybrids of any of the four PBCs in the upscaling pipeline fail on any step, for example, lower than expected multiplication rates or unforeseen agronomic barriers, then further selections from ongoing breeding are needed to replace earlier candidates.

In conclusion, the breeding foundations have been laid well for switchgrass, miscanthus, willow and poplar owing to public funding over the long time periods necessary. Improved cultivars or genotypes are available that could be scaled up over a few years; if real sustained market opportunities emerged in response to sustained favourable policies and industrial market pull. The potential contributions of growing and using these PBCs for socioeconomic and environmental benefits are clear, but how farmers and others in commercial value chains are rewarded for mass‐scale deployment, as is necessary, is not obvious at present. Therefore, mass‐scale deployment of these lignocellulose crops needs developments outside the breeding arenas to drive breeding activities more rapidly and extensively.

## 8 Uncited reference

Huang et al. (2019).

## CONFLICT OF INTEREST

The authors declare that progress reported in this paper, which includes input from industrial partners, is not biased by their business interests.

9
